# N‐Containing Carbon‐Coated *β*‐Si_3_N_4_ Enhances Si Anodes for High‐Performance Li‐Ion Batteries

**DOI:** 10.1002/advs.202301218

**Published:** 2023-05-11

**Authors:** Rahmandhika Firdauzha Hary Hernandha, Bharath Umesh, Purna Chandra Rath, Le Thi Thu Trang, Ju‐Chao Wei, Yu‐Chun Chuang, Ju Li, Jeng‐Kuei Chang

**Affiliations:** ^1^ Department of Materials Science and Engineering National Yang Ming Chiao Tung University 1001 University Road Hsinchu 30010 Taiwan; ^2^ Institute of Materials Science and Engineering National Central University 300 Zhong‐Da Road Taoyuan 32001 Taiwan; ^3^ Materials Science Group, National Synchrotron Radiation Research Center Super Energy Materials Inc. 99‐1 Xiyuan Road Taoyuan 32057 Taiwan; ^4^ National Synchrotron Radiation Research Center Hsin‐Ann Road Hsinchu 30076 Taiwan; ^5^ Department of Nuclear Science and Engineering and Department of Materials Science and Engineering Massachusetts Institute of Technology 77 Massachusetts Avenue Cambridge MA 02139 USA; ^6^ Department of Chemical Engineering Chung Yuan Christian University 200 Chung Pei Road Taoyuan 32023 Taiwan

**Keywords:** beta silicon nitride fraction, carbon coating, lithium nitride, operando X‐ray diffraction, silicon nitride allotrope

## Abstract

The lithiation/delithiation properties of *α*‐Si_3_N_4_ and *β*‐Si_3_N_4_ are compared and the carbon coating effects are examined. Then, *β*‐Si_3_N_4_ at various fractions is used as the secondary phase in a Si anode to modify the electrode properties. The incorporated *β*‐Si_3_N_4_ decreases the crystal size of Si and introduces a new N—Si—O species at the *β*‐Si_3_N_4_/Si interface. The nitrogen from the milled *β*‐Si_3_N_4_ diffuses into the surface carbon coating during the carbonization heat treatment, forming pyrrolic nitrogen and C—N—O species. The synergistic effects of combining *β*‐Si_3_N_4_ and Si phases on the specific capacity are confirmed. The operando X‐ray diffraction and X‐ray photoelectron spectroscopy data indicate that *β*‐Si_3_N_4_ is partially consumed during lithiation to form a favorable Li_3_N species at the electrode. However, the crystalline structure of the hexagonal *β*‐Si_3_N_4_ is preserved after prolonged cycling, which prevents electrode agglomeration and performance deterioration. The carbon‐coated *β*‐Si_3_N_4_/Si composite anode shows specific capacities of 1068 and 480 mAh g^−1^ at 0.2 and 5 A g^−1^, respectively. A full cell consisting of the carbon‐coated *β*‐Si_3_N_4_/Si anode and a LiNi_0.8_Co_0.1_Mn_0.1_O_2_ cathode is constructed and its properties are evaluated. The potential of the proposed composite anodes for Li‐ion battery applications is demonstrated.

## Graphical abstract

The superior charge/discharge properties of *β*‐Si_3_N_4_ over *α*‐Si_3_N_4_ are confirmed. *β*‐Si_3_N_4_ is partially consumed during lithiation to form a protective Li_3_N layer. With incorporated *β*‐Si_3_N_4_ in a Si‐based anode, Li_15_Si_4_ formation/dissolution upon charge/discharge is highly reversible. The crystallinity of *β*‐Si_3_N_4_ is preserved after cycling, suppressing electrode deterioration. Combining *β*‐Si_3_N_4_ and Si phases results in a clear synergy in specific capacities.

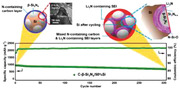

## 1 Introduction

Advancements in Li‐ion battery (LIB) technology are driven by the rising demand for portable electronic devices, electric vehicles, and large‐scale energy storage, especially for renewable sources. Higher energy density is the main target for LIBs.^[^ ^1^, ^2^ ^]^ Graphitized carbon is commonly used as a negative electrode in commercial LIBs. However, its limited capacity (theoretically, 372 mAh g^−1^) does not meet the escalating application requirements. Improvement that can lead to better electrode capacity is thus urgently needed. As an alternative, Si‐based anodes are highly promising for meeting the required properties.^[^ ^3^, ^4^ ^]^ Si is an abundant material (28% of the Earth's crust by mass) with a high theoretical capacity of ≈3579 mAh g^−1^, desirable redox potential, low cost, and non‐toxicity.^[^ ^5^, ^6^, ^7^ ^]^ Despite these appealing features, Si anodes suffer from large volume variation (>300%) during cycling, which leads to electrode mechanical degradation and an unstable solid‐electrolyte interphase (SEI). Various strategies have been developed to fabricate diverse nano‐architectures, such as carbon‐coated,^[^ ^8^ ^]^ core‐shell/yolk‐shell,^[^ ^9^ ^]^ hollow,^[^ ^10^ ^]^ porous,^[^ ^11^ ^]^ fibrous,^[^ ^12^ ^]^ tubular,^[^ ^13^ ^]^ and micropillar^[^ ^14^ ^]^ Si‐based composites, for improving cycling stability. However, the long‐term cyclability of Si‐based anodes is not yet satisfactory.^[^ ^15^ ^]^ A more robust and reliable Si electrode with a high capacity, superior rate capability, and reasonable cycle life is desirable.

Introducing a secondary phase to alleviate the Si volume change during charge–discharge cycling has been proven to be an effective strategy for reducing stress localization and thus increasing electrode cycling stability.^[^ ^16^ ^]^ Accordingly, Si—Cu,^[^ ^17^ ^]^ Si—Fe,^[^ ^18^ ^]^ Si—Ni,^[^ ^19^ ^]^ Si—Ti,^[^ ^20^ ^]^ Si—Fe—Cu,^[^ ^21^ ^]^ Si—Fe—Ti,^[^ ^22^ ^]^ and Si—Ni—Ti^[^ ^23^ ^]^ composite anodes have been developed. However, some secondary phases vigorously participate in the lithiation/delithiation reactions and thus undergo substantial volume expansion/contraction,^[^ ^18^, ^20^, ^21^, ^23^ ^]^ leading to structural deterioration upon cycling. Furthermore, the introduced silicide phases usually have low Li^+^ conductivity,^[^ ^23^, ^24^ ^]^ which results in limited redox kinetics. In addition, the reduced transition metals in the electrodes after lithiation could catalyze electrolyte decomposition^[^ ^25^, ^26^, ^27^ ^]^ and/or change the SEI chemistry and nanostructure.^[^ ^28^ ^]^ This may impede Li^+^ transport and ultimately degrade electrode performance. Therefore, the development of a better secondary phase that is mechanically and dimensionally stable and can help the formation of a suitable SEI and effective Li^+^ transport pathways in the Si electrode is important.

A few studies have attempted to incorporate crystalline silicon nitride (Si_3_N_4_) into Si‐based anodes.^[^ ^29^, ^30^, ^31^, ^32^ ^]^ Crystalline Si_3_N_4_ has excellent strength and toughness,^[^ ^33^, ^34^ ^]^ which are beneficial for mechanically stabilizing Si electrodes. These studies indicated that crystalline Si_3_N_4_ is inactive in the electrodes.^[^ ^29^, ^30^, ^31^, ^32^ ^]^ In other words, it neither participated in the lithiation/delithiation reactions nor contributed to the measured capacity. Instead, the Si_3_N_4_ served as a framework to prevent the Si electrodes from mechanical collapse. Zhang et al. synthesized a composite that consisted of Si particles and *α*‐Si_3_N_4_ whiskers and found that the inactive *α*‐Si_3_N_4_ can be a buffer matrix that supports active Si particles and prevents them from aggregating.^[^ ^29^ ^]^ Kim et al. prepared a 3D flexible Si composite anode that consisted of core (Si)‐shell (*α*‐Si_3_N_4_) particles and carbon nanofibers.^[^ ^30^ ^]^ The Si_3_N_4_ shells effectively maintained the structural stability of the electrode. Xiao et al. fabricated an egg‐like Si/*α*‐Si_3_N_4_/C composite anode and reported that a strong and tough *α*‐Si_3_N_4_ eggshell layer can restrain the structural collapse and facilitate the Li^+^ transport within the electrode.^[^ ^31^ ^]^ Of note, crystalline Si_3_N_4_ exists in two allotropes, namely *α*‐Si_3_N_4_ and *β*‐Si_3_N_4_, which exhibit distinct physicochemical properties.^[^ ^35^ ^]^ Specifically, *β*‐Si_3_N_4_ has a higher bulk density than that of *α*‐Si_3_N_4_.^[^ ^36^ ^]^ In addition, *β*‐Si_3_N_4_ is chemically and thermally more stable than *α*‐Si_3_N_4_.^[^ ^37^ ^]^ *α*‐Si_3_N_4_ has a trigonal structure, where each nitrogen atom is bonded to three silicon atoms in a distorted trigonal configuration and each silicon atom is tetrahedrally bonded to four nitrogen atoms. In contrast, *β*‐Si_3_N_4_ has a phenacite structure that consists of a trigonal arrangement of silicon atoms bonded to nitrogen atoms. When viewed along the *c*‐axis looking down onto the basal plane of *β*‐Si_3_N_4_, a hexagonal arrangement of void channels is visible.^[^ ^38^ ^]^ The unique crystal structure of *β*‐Si_3_N_4_ could favor Li^+^ transport and lead to superior electrochemical properties. However, to the best of our knowledge, there have been no studies on the application of *β*‐Si_3_N_4_ to LIBs, a topic that is worth studying. The fraction and distribution of crystalline Si_3_N_4_ are considered to crucially impact Si anode performance. The synergistic effects between Si_3_N_4_ and Si phases have not yet been explored; they need to be examined in detail to further understand the underlying mechanism. It is worth mentioning that a facile, energy‐efficient, and scalable synthesis route is desirable for practical LIB applications. A previously reported 3D Si core/*α*‐Si_3_N_4_ shell/carbon nanofiber sample was fabricated using electrospinning combined with calcination at 1200 °C.^[^ ^30^ ^]^ The egg‐like Si/*α*‐Si_3_N_4_/C composite was produced via a two‐step reaction, including a nitrification process at 1275 °C and a chemical‐vapor‐deposition carbon‐coating process at 850 °C.^[^ ^31^ ^]^ A more convenient and cost‐effective method for constructing the desired Si/Si_3_N_4_ composite is our target.

In this study, the lithiation/delithiation properties of *α*‐Si_3_N_4_ and *β*‐Si_3_N_4_ are systematically compared. A carbon coating is deposited on both kinds of particles to enhance electrochemical performance. Then, *β*‐Si_3_N_4_ and Si at various phase fractions are integrated and their synergistic effects on electrode capacity and rate capability are investigated. The microstructure, crystallinity, chemical composition, and SEI chemistry of various *β*‐Si_3_N_4_/Si electrodes are studied to explain the synergy. We confirm that the *β*‐Si_3_N_4_ is not inactive. Operando X‐ray diffraction (XRD) is performed to examine the role of *β*‐Si_3_N_4_ in altering the electrode lithiation/delithiation behavior. The carbon‐coated *β*‐Si_3_N_4_/Si is mixed with artificial graphite (AG) to fabricate a composite anode. Full cells using LiNi_0.8_Co_0.1_Mn_0.1_O_2_ cathodes are constructed and their charge–discharge performance is evaluated. The incorporation of the proposed carbon‐coated *β*‐Si_3_N_4_ is shown to significantly enhance the rate capability and cyclability of Si anodes for next‐generation LIBs.

## 2 Results and Discussion

**Figure 1:**
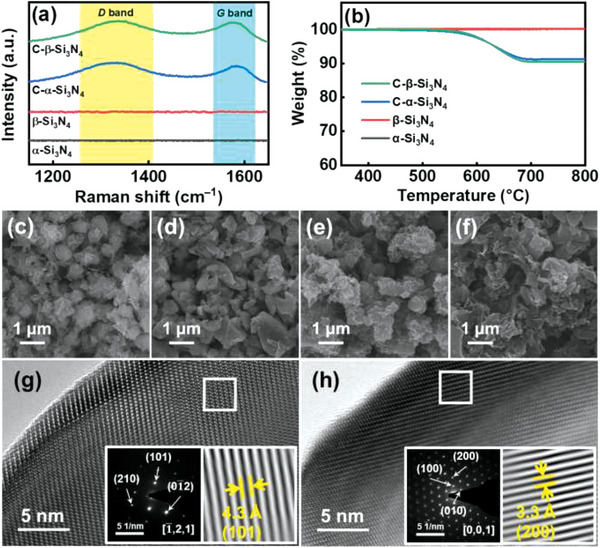
a) Raman spectra, b) TGA data, SEM images of c) *α*‐Si_3_N_4_, d) *β*‐Si_3_N_4_, e) C‐*α*‐Si_3_N_4_, and f) C‐*β*‐Si_3_N_4_, and high‐resolution TEM images of g) *α*‐Si_3_N_4_ and h) *β*‐Si_3_N_4_.

The crystallinity of the carbon‐coated *α*‐Si_3_N_4_ (denoted as C‐*α*‐Si_3_N_4_) and carbon‐coated *β*‐Si_3_N_4_ (denoted as C‐*β*‐Si_3_N_4_) was examined using XRD; the obtained diffraction patterns are shown in Figure S1, Supporting Information. The peaks at 20.6°, 22.9°, 26.5°, 31.0°, 34.6°, and 35.3° are respectively indexed as (101), (110), (200), (201), (102), and (210) plane diffraction of trigonal *α*‐Si_3_N_4_ (JCPDS‐41‐0360). The peaks at 13.4°, 23.4°, 27.1°, 33.7°, 36.1°, and 41.4° are associated with the (100), (110), (200), (101), (210), and (201) plane diffraction of hexagonal *β*‐Si_3_N_4_ (JCPDS‐33‐1160). The *α* phase was determined to have the lattice parameters *a* = *b* = 7.766(2) Å and *c* = 5.615(3) Å, whereas the *β* phase had the lattice parameters *a* = *b* = 7.586(2) Å and *c* = 2.902(1) Å. In each Si_3_N_4_ sample, the other allotrope appeared as a minor fraction; specifically, the *α*‐Si_3_N_4_ sample had ≈2% *β*‐Si_3_N_4_ and the *β*‐Si_3_N_4_ sample had ≈7% *α*‐Si_3_N_4_. No other impurity phases were detected. Due to the low crystallinity of the carbon layers, no distinct diffraction signal was observed. **Figure** **1**a shows the Raman spectra of the *α*‐Si_3_N_4_, *β*‐Si_3_N_4_, C‐*α*‐Si_3_N_4_, and C‐*β*‐Si_3_N_4_ samples. Both carbon‐coated samples exhibit a *D*‐band signal at ≈1345 cm^−1^ and a *G*‐band signal at ≈1595 cm^−1^. The former is associated with imperfect carbon bonding and the latter originates from the Raman‐allowed in‐plane vibration of sp^2^ carbon. The *D*‐to‐*G*‐band intensity ratio (*I*_D_/*I*_G_) is a critical index that reflects the graphitization degree of carbon materials. The *I*_D_/*I*_G_ ratios of C‐*α*‐Si_3_N_4_ and C‐*β*‐Si_3_N_4_ are ≈1.1, indicating that the deposited carbon was amorphous.^[^ ^39^, ^40^ ^]^ The carbon content of the samples was quantitatively evaluated with TGA; the data are shown in Figure 1b. Both the C‐*α*‐Si_3_N_4_ and C‐*β*‐Si_3_N_4_ samples underwent clear weight loss at around 550–680 °C, which was associated with the burnout of the carbon layers. The data indicate that *α*‐Si_3_N_4_ and *β*‐Si_3_N_4_ have great thermal stability and that the carbon content of both coated samples is approximately 10 wt.%. Figure 1c–f shows Scanning electron microscopy (SEM) images of *α*‐Si_3_N_4_, *β*‐Si_3_N_4,_ C‐*α*‐Si_3_N_4,_ and C‐*β*‐Si_3_N_4_ powders, respectively. Irregularly granular morphologies were observed for all samples. Figure 1g,h respectively shows high‐resolution lattice images of *α*‐Si_3_N_4_ and *β*‐Si_3_N_4_ particles, which reveal that both allotropes are highly crystalline. The lattice fringes with spacings of 4.3 and 3.3 Å are associated with the (101) and (200) plane distances of the *α*‐Si_3_N_4_ and *β*‐Si_3_N_4_ crystals, respectively. The electron diffraction patterns shown in the figure insets confirm the phase identification results obtained from XRD.

**Figure 2:**
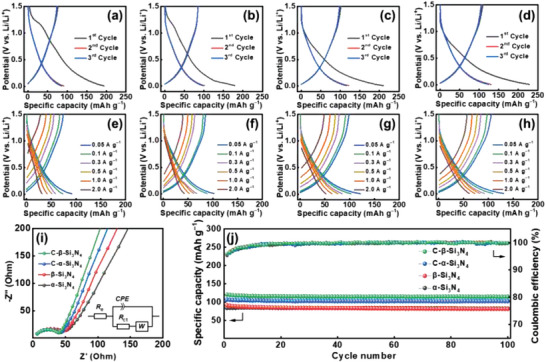
Charge–discharge curves of a,e) *α*‐Si_3_N_4_, b,f) *β*‐Si_3_N_4_, c,g) C‐*α*‐Si_3_N_4_, and d,h) C‐*β*‐Si_3_N_4_ electrodes. i) EIS data of various electrodes and equivalent circuits used for data fitting. j) Cycling stability of various electrodes measured at 0.05 A g^−1^.

**Figure** **2**a–d shows the initial charge–discharge curves of various electrodes measured at a current rate of 0.05 A g^−1^. The initial Coulombic efficiency (CE) values of the *α*‐Si_3_N_4_, *β*‐Si_3_N_4_, C‐*α*‐Si_3_N_4,_ and C‐*β*‐Si_3_N_4_ electrodes are 43%, 52%, 54%, and 57%, respectively. The efficiency loss is ascribed to SEI formation and the irreversible trapping of Li^+^ ions within the electrodes. The carbon coating improved electron and Li^+^ conduction, thus increasing CE and the measured capacities. The intrinsic electronic conductivity values of *α*‐Si_3_N_4_ and *β*‐Si_3_N_4_ are ≈10^−13^ S cm^−1^.^[^ ^41^, ^42^ ^]^ After carbon coating, the values increase to 1.02 × 10^−1^ and 1.07 × 10^−1^ S cm^−1^, respectively (Table S1, Supporting Information). Figure 2e–h shows the electrode charge–discharge profiles recorded at various current rates after two conditioning cycles. The reversible capacities obtained at 0.05 A g^−1^ are 80, 91, 109, and 118 mAh g^−1^ for the *α*‐Si_3_N_4_, *β*‐Si_3_N_4_, C‐*α*‐Si_3_N_4_, and C‐*β*‐Si_3_N_4_ electrodes, respectively. With increasing current rate, the specific capacities decreased, as shown in Table S2, Supporting Information. The capacities decreased to 31, 42, 52, and 62 mAh g^−1^, respectively, at a specific current of 2 A g^−1^, corresponding to 39%, 46%, 48%, and 53% of the capacities found at 0.05 A g^−1^. This is the first electrochemical performance comparison between two Si_3_N_4_ allotropes. The superior charge–discharge properties of *β*‐Si_3_N_4_ could be associated with *β*‐Si_3_N_4_ having continuous *c*‐axis channels that are approximately 300 pm in diameter, which could benefit Li^+^ transport owing to the wide space between basal layers constituted by the linking of six eight‐member Si—N rings.^[^ ^33^, ^38^ ^]^ In contrast, for *α*‐Si_3_N_4_, because of the existence of a *c* glide plane, the continuous channels are interrupted, and thus a series of interstices form,^[^ ^33^ ^]^ which is unfavorable for Li^+^ conduction. Figure 2i shows the electrochemical impedance spectroscopy (EIS) data of various electrodes acquired after two conditioning cycles. The Nyquist spectra consist of a semicircle at high frequency and a sloping line at low frequency, which can be characterized by the equivalent circuit shown in the figure inset, where *R*_e_, *R*_ct_, CPE, and *W* represent the electrolyte resistance, charge transfer resistance, interfacial constant‐phase element, and Warburg impedance associated with Li^+^ diffusion within the electrode, respectively.^[^ ^43^ ^]^ The *R*_ct_ values derived from the data fitting are 44, 39, 34, and 31 Ω for the *α*‐Si_3_N_4_, *β*‐Si_3_N_4_, C‐*α*‐Si_3_N_4_, and C‐*β*‐Si_3_N_4_ electrodes, respectively. The apparent Li^+^ diffusion coefficient (*D*_Li_^+^_(EIS)_) can be estimated from the EIS sloping line in the low‐frequency region.^[^ ^44^ ^]^ The calculated *D*_Li_^+^_(EIS)_ values for the four electrodes are 2.6, 3.1, 6.1, and 6.9 × 10^−14^ cm^2^ s^−1^, respectively. Both the *R*_ct_ and *D*_Li_^+^ data support C‐*β*‐Si_3_N_4_ having the best high‐rate performance among the electrodes. Figure 2j shows the cycling stability data of the electrodes measured at 0.05 A g^−1^. All the electrodes are highly stable over cycling, with the capacity retention being above 95% and the saturated CE values being above 99.9%. Because C‐*β*‐Si_3_N_4_ had the best performance, it was used for further analyses.

**Figure 3:**
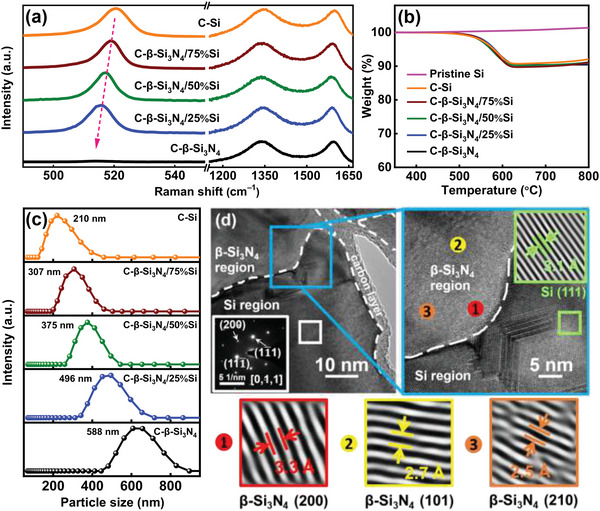
a) Raman spectra, b) TGA data, and c) particle size distribution of various samples. d) High‐resolution TEM images of C‐*β*‐Si_3_N_4_/50%Si sample.

Next, we attempted to integrate *β*‐Si_3_N_4_ and Si with various weight ratios using high‐energy ball milling. **Figure** **3**a shows the Raman spectra of various samples. The peak, which is related to the vibration band of polycrystalline Si,^[^ ^32^ ^]^ shifts from 520 to 515 cm^−1^ with increasing *β*‐Si_3_N_4_ fraction. This is associated with Si lattice distortion and the formation of an interface in the composites.^[^ ^32^, ^45^ ^]^ Figure 3a also shows the carbon *D* and *G* bands for all the C‐*β*‐Si_3_N_4_/Si samples, confirming that the carbon coating was successfully deposited. According to the TGA data in Figure 3b, the carbon content of all the samples was approximately ≈10 wt.%. Figure 3c shows the results of the dynamic light scattering (DLS) measurements. According to the particle size distribution profiles, the *D*_50_ values of C‐*β*‐Si_3_N_4_, C‐*β*‐Si_3_N_4_/25%Si, C‐*β*‐Si_3_N_4_/50%Si, C‐*β*‐Si_3_N_4_/75%Si, and C—Si are 588, 496, 375, 307, and 210 nm, respectively. It is noted that only a single DLS peak was observed for all the samples. These results suggest that the *β*‐Si_3_N_4_ and Si phases were well integrated, creating a homogeneous composite. Figure S2, Supporting Information, shows the XRD patterns of various samples, confirming the dual‐phase nature of the *β*‐Si_3_N_4_/Si composites without the formation of new compounds after mixing. Table S3, Supporting Information, shows the tap densities of the samples. As shown, a higher *β*‐Si_3_N_4_ fraction led to higher density. A denser material is essential for making a more compact electrode, which is favorable for electrode volumetric performance.^[^ ^46^ ^]^ Figure 3d shows high‐resolution transmission electron microscopy (TEM) images of the C‐*β*‐Si_3_N_4_/50%Si sample. As shown, the carbon coating is about ≈5 nm in thickness and the two phases are integrated. The phase boundary between Si and *β*‐Si_3_N_4_ is marked in the figure. The lower part of the particle is a Si phase domain, as confirmed by the electron diffraction pattern shown in the figure inset. The observed *d* spacing of 3.1 Å is attributed to the Si (111) plane distance. The upper part of the particle is identified as polycrystalline *β*‐Si_3_N_4_. The three distinct positions marked in the figure have *d* spacing values of 3.3, 2.7, and 2.5 Å, corresponding to the (200), (101), and (210) planes of *β*‐Si_3_N_4_, respectively.

The corrosion resistance of the C‐*β*‐Si_3_N_4_, C‐*β*‐Si_3_N_4_/50%Si, and C—Si to HF were evaluated. The samples were immersed in 25 mm HF aqueous solution at 25 °C for 1 h, and the weight loss data are shown in Figure S3, Supporting Information. It is confirmed that the dissolution rate of C‐*β*‐Si_3_N_4_ is much lower than that of C—Si.

**Figure 4:**
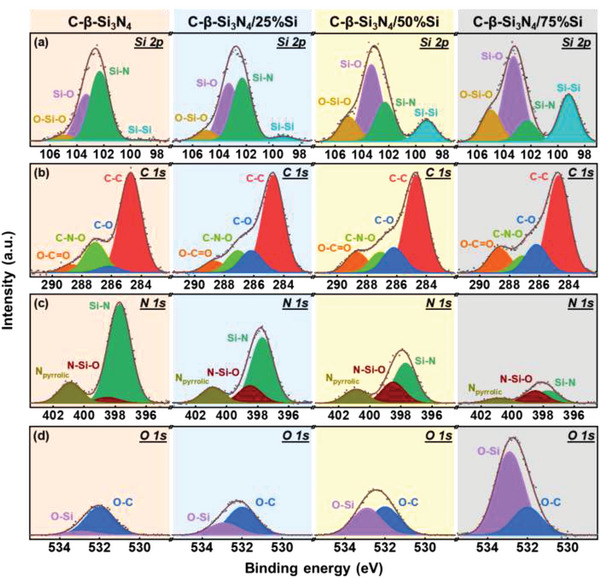
XPS a) Si 2p, b) C 1s, c) N 1s, and d) O 1s spectra of various samples.

**Figure** **4**a shows the X‐ray photoelectron spectroscopy (XPS) Si 2p spectra of the C‐*β*‐Si_3_N_4_, C‐*β*‐Si_3_N_4_/25%Si, C‐*β*‐Si_3_N_4_/50%Si, and C‐*β*‐Si_3_N_4_/75%Si samples, respectively. The spectra can be split into four peaks: Si—Si at 99.2 eV, Si—N at 102.3 eV, Si—O at 103.3 eV, and O—Si—O at 105.0 eV.^[^ ^8^, ^30^ ^]^ With increasing Si phase fraction, the Si—Si, Si—O, and O—Si—O intensities monotonically increase, indicating that the Si (rather than *β*‐Si_3_N_4_) particle surface is relatively oxygen‐enriched. The C 1s spectra of the samples are shown in Figure 4b. Higher C—O (at 286.2 eV) and O—C=O (at 288.7 eV) concentrations are found for the sample with a higher Si phase content. It is noted that the C—N—O bonds become intensified when the *β*‐Si_3_N_4_ fraction increases. The N 1s spectra presented in Figure 4c can be deconvoluted into three components. The distinctive peaks at 397.7, 398.5, and 400.9 eV are associated with Si—N, N—Si—O, and pyrrolic nitrogen, respectively. The existence of pyrrolic nitrogen implies that the nitrogen from the milled *β*‐Si_3_N_4_ could diffuse into the carbon layers. The N—Si—O is supposed to be a new species that formed at the *β*‐Si_3_N_4_/Si interface. Figure 4d shows the O 1s spectra, which are split into two peaks, namely O—C and O—Si peaks at 532.0 and 532.9 eV, respectively. The intensity of the latter peak increases with Si phase fraction, which can be explained by Si being intrinsically active and easily oxidized^[^ ^47^, ^48^ ^]^ during synthesis (see Experimental section for details).

To study the electrochemical properties of various electrodes, CV measurements were performed. Figure S4a–e, Supporting Information, shows the obtained CV curves of the C‐*β*‐Si_3_N_4_, C‐*β*‐Si_3_N_4_/25%Si, C‐*β*‐Si_3_N_4_/50%Si, C‐*β*‐Si_3_N_4_/75%Si, and C—Si electrodes, respectively. The cathodic peaks at ≈1.2 V for all electrodes can be assigned to the reductive decomposition of the electrolyte and the formation of SEI layers.^[^ ^49^, ^50^ ^]^ For the C‐*β*‐Si_3_N_4_ electrode, there is a clear redox current below ≈0.5 V, indicating that *β*‐Si_3_N_4_ is not completely inert. Some conversion and/or intercalation reactions may be involved (discussed later). However, bulk transformation from *β*‐Si_3_N_4_ to Si did not happen because no visible Si alloying/dealloying CV characteristics were found. The XRD and XPS data in Figure S5, Supporting Information, further support that there is no formation of Si phase after the CV scans. As shown, with increasing Si phase fraction in the electrodes, the redox signals of Si become more pronounced. The reduction peaks at ≈0.2 V and below 0.1 V can be ascribed to the evolution of various Li‐Si alloy phases.^[^ ^8^ ^]^ Upon the anodic scan, two distinct peaks emerged at 0.38 and 0.51 V, which correspond to the phase transition from Li_15_Si_4_ to amorphous Li*_x_* Si and that from amorphous Li*_x_* Si to Si, respectively.^[^ ^51^ ^]^ During consecutive scans, the CV current increased, reflecting the electrode activation process toward lithiation/delithiation.^[^ ^52^, ^53^ ^]^

**Figure 5:**
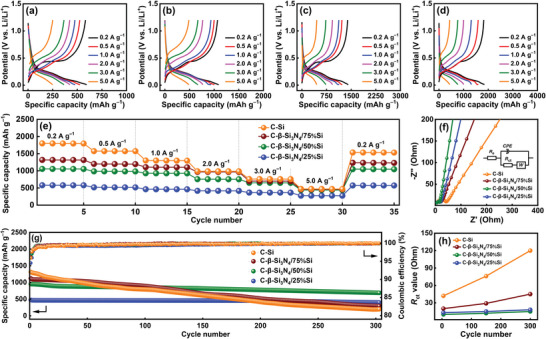
Charge–discharge profiles of a) C‐*β*‐Si_3_N_4_/25%Si, b) C‐*β*‐Si_3_N_4_/50%Si, c) C‐*β*‐Si_3_N_4_/75%Si, and d) C—Si electrodes measured at various rates. e) Comparative rate performance of various electrodes. f) EIS spectra of various electrodes. g) Cycling stability of various electrodes measured at 1 A g^−1^. h) Variation of *R*_ct_ values of various electrodes with respect to charge–discharge cycle number.

Figure S6a–d, Supporting Information, shows the initial three charge–discharge cycles of various electrodes measured at 0.2 A g^−1^. The first‐cycle CE values of the C‐*β*‐Si_3_N_4_/25%Si, C‐*β*‐Si_3_N_4_/50%Si, C‐*β*‐Si_3_N_4_/75%Si, and C—Si electrodes are 65%, 78%, 83%, and 86%, respectively. Some irreversible redox reactions were involved for *β*‐Si_3_N_4_, leading to a lower initial CE for the electrode with a higher *β*‐Si_3_N_4_ fraction. As shown in Figure S6, Supporting Information, when the Si fraction exceeded 50%, the delithiation (defined as discharge in this study) capacity decay upon cycling was pronounced. This suggests that *β*‐Si_3_N_4_ can help stabilize electrode performance. **Figure** **5**a–d shows the charge–discharge profiles of the electrodes measured at various rates after two conditioning cycles. The reversible capacities obtained at 0.2 A g^−1^ are 583, 1068, 1390, and 1820 mAh g^−1^ for the C‐*β*‐Si_3_N_4_/25%Si, C‐*β*‐Si_3_N_4_/50%Si, C‐*β*‐Si_3_N_4_/75%Si, and C—Si electrodes, respectively. As shown in Figure 5e and **Table** **1**, the specific capacities of these electrodes decrease to 250, 488, 452, and 437 mAh g^−1^, respectively, at a specific current of 5 A g^−1^, corresponding to 43%, 46%, 33%, and 24% retention compared to the capacities found at 0.2 A g^−1^. Figure 5f shows the EIS data of the electrodes acquired after the conditioning cycles. The *R*_ct_ values are 13, 10, 20, and 42 Ω for C‐*β*‐Si_3_N_4_/25%Si, C‐*β*‐Si_3_N_4_/50%Si, C‐*β*‐Si_3_N_4_/75%Si, and C—Si, respectively (**Table** **2**). The Li^+^ transport properties within the electrodes were evaluated using the galvanostatic intermittent titration technique (GITT).^[^ ^54^ ^]^ The results are shown in Figure S7, Supporting Information, and the calculated average *D*_Li_^+^ values (*D*_Li_^+^_(GITT)_) are summarized in Table 2. As shown, the C‐*β*‐Si_3_N_4_/50%Si electrode has the highest *D*_Li_^+^_(GITT)_ values of 4.6 and 5.1× 10^−10^ cm^2^ s^−1^, for lithiation and delithiation, respectively. The favorable *R*_ct_ and *D*_Li_^+^_(GITT)_ values explain the optimal rate capability of the C‐*β*‐Si_3_N_4_/50%Si electrode. The expected capacities of various C‐*β*‐Si_3_N_4_/Si composite anodes can be calculated from the linear combination of the measured capacities of the C‐*β*‐Si_3_N_4_ and C—Si electrodes, as shown in Table S4, Supporting Information. As shown, the real capacities of C‐*β*‐Si_3_N_4_/Si composite anodes are higher than the expected values, confirming that synergistic effects happen, especially at high rates. It is noted that the strongest synergy is found for the C‐*β*‐Si_3_N_4_/50%Si electrode. The Si phase was modified (Figure 3a) by the incorporation of *β*‐Si_3_N_4_. However, a high *β*‐Si_3_N_4_ fraction led to a lower electronic conductivity of the composite (Table S1, Supporting Information). Moreover, the created interface between Si and *β*‐Si_3_N_4_ may favor Li^+^ transport. There is thus an optimal *β*‐Si_3_N_4_‐to‐Si ratio that maximizes electrode performance.

**Table 1:** Reversible specific capacities of C‐*β*‐Si_3_N_4_/25%Si, C‐*β*‐Si_3_N_4_/50%Si, C‐*β*‐Si_3_N_4_/75%Si, and C—Si electrodes measured at various specific currents

Current rate [A g^−1^]	C‐*β*‐Si_3_N_4_/25%Si [mAh g^−1^]	C‐*β*‐Si_3_N_4_/50%Si [mAh g^−1^]	C‐*β*‐Si_3_N_4_/75%Si [mAh g^−1^]	C—Si [mAh g^−1^]
0.2	583	1068	1390	1820
0.5	525	1000	1218	1591
1	476	935	1124	1311
2	424	769	930	1001
3	365	670	728	765
5	250	488	452	437
High rate retention^a)^	43%	46%	33%	24%

^a)^ a comparison between reversible capacities at 5 and 0.2 A g ^−1^.

**Table 2:** *R*_ct_ and *D*_Li_^+^_(GITT)_ values of various electrodes measured after conditioning cycles

Samples	*R*_ct_ [Ω]	*D*_Li_^+^_(GITT)_ for lithiation/delithiation [× 10^−10^ cm^2^ s^−1^]
C‐*β*‐Si_3_N_4_/25%Si	13	3.7/4.8
C‐*β*‐Si_3_N_4_/50%Si	10	4.6/5.1
C‐*β*‐Si_3_N_4_/75%Si	20	3.1/4.2
C—Si	42	2.5/3.3

Figure 5g shows the cycling stability data of various electrodes measured at 1 A g^−1^. The steady CE values are 99.9%, 99.9%, 99.8%, and 99.7% for the C‐*β*‐Si_3_N_4_/25%Si, C‐*β*‐Si_3_N_4_/50%Si, C‐*β*‐Si_3_N_4_/75%Si, and C—Si electrodes, respectively. After 300 charge–discharge cycles, the electrodes retained 84%, 75%, 30%, and 15% of their initial capacities, respectively. A satisfactory cycling stability can be achieved with a Si fraction of up to 50%. The specific capacity of 700 mAh g^−1^ for C‐*β*‐Si_3_N_4_/50%Si is the highest value among the electrodes studied after 300 cycles. As shown in Table S5, Supporting Information, this durability is comparable to the best performance reported in the literature. The impedance evolution of the electrodes after cycling was also investigated (see Figure S8, Supporting Information). As shown in Figure 5h, the *R*_ct_ values clearly increase upon cycling when the Si phase fraction is more than 50%. The postmortem SEM images of various electrodes after cycling are shown in Figure S9, Supporting Information. The morphologies of the C‐*β*‐Si_3_N_4_/75%Si and C—Si electrodes were clearly distorted compared to those of the pristine electrodes. The Si particles significantly expanded and agglomerated. Moreover, both electrodes were covered by thick SEI layers, leading to increased *R*_ct_ and capacity deterioration. In contrast, an adequate fraction of *β*‐Si_3_N_4_ (i.e., ≥50%) is beneficial for preserving the electrode structural stability during repeated lithiation/delithiation. Figure S10, Supporting Information, shows the cross‐section SEM images of the C‐*β*‐Si_3_N_4_/50%Si and C—Si electrodes before and after 25 charge–discharge cycles. Much less volume expansion and better integrity of the former electrode are confirmed.

**Figure 6:**
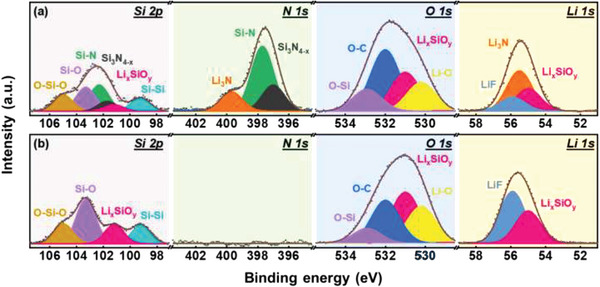
XPS Si 2p, N 1s, O 1s, and Li 1s spectra of a) C‐*β*‐Si_3_N_4_/50%Si and b) C—Si electrodes after conditioning cycles.

To gain more insight into the SEI chemistry, XPS analyses were performed for the C‐*β*‐Si_3_N_4_/50%Si and C—Si electrodes after two conditioning cycles. As shown by the Si 2p orbital data in **Figure** **6**, a new Si_3_N_4‐_ *_x_* species formed for the C‐*β*‐Si_3_N_4_/50%Si electrode, suggesting that the nitrogen in *β*‐Si_3_N_4_ was consumed. In addition, Li*_x_* SiO*_y_* was found to be a compound in both SEI layers. The N 1s spectrum indicates the formation of Li_3_N^[^ ^55^, ^56^ ^]^ on the C‐*β*‐Si_3_N_4_/50%Si electrode. Upon lithiation, Li^+^ ions reacted with the nitrogen in *β*‐Si_3_N_4_ to form Li_3_N, leaving behind Si_3_N_4‐_ *_x_*. Of note, Li_3_N is a highly favorable SEI component that can provide high mechanical robustness, great passivation ability, and high Li^+^ conductivity.^[^ ^57^, ^58^, ^59^ ^]^ In the O 1s spectra, four constituents were observed, namely Li—O, Li*_x_* SiO*_y_*, O—C, and O—Si. The C—Si surface is relatively Li—O‐ and Li*_x_* SiO*_y_*‐enriched, implying that the SEI layer is thicker than that on the C‐*β*‐Si_3_N_4_/50%Si electrode. Based on the Li 1s spectra, the SEI on C‐*β*‐Si_3_N_4_/50%Si has high Li_3_N content, whereas that of C—Si is composed of LiF and Li*_x_* SiO*_y_*. The much higher Li^+^ conductivity of Li_3_N^[^ ^60^ ^]^ compared to that of LiF explains the superior rate capability of the C‐*β*‐Si_3_N_4_/50%Si electrode. Figure S11, Supporting Information, shows the XPS analysis data of the C‐*β*‐Si_3_N_4_/50%Si electrode after 300 charge–discharge cycles. As shown, the surface chemical composition is close to that of the electrode before cycling (Figure 6a). This indicates that the Li_3_N‐containing SEI, derived from *β*‐Si_3_N_4_, is stable and robust against long cycling.

**Figure 7:**
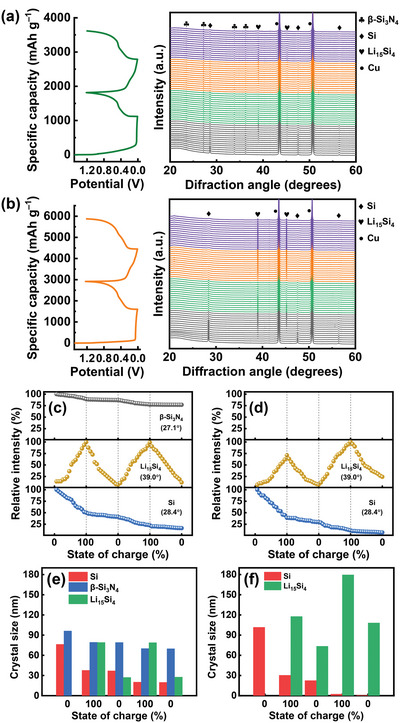
Operando XRD data of a) C‐*β*‐Si_3_N_4_/50%Si and b) C—Si electrodes during initial two charge–discharge cycles. Relative peak intensity versus state of charge of c) C‐*β*‐Si_3_N_4_/50%Si and d) C—Si electrodes. Crystal size variation of Si, *β*‐Si_3_N_4_, and Li_15_Si_4_ in e) C‐*β*‐Si_3_N_4_/50%Si and f) C—Si electrodes.

**Figure** **7**a,b shows operando XRD data of the C‐*β*‐Si_3_N_4_/50%Si and C—Si electrodes, respectively, during the initial two charge–discharge cycles. As shown in Figure 7c, the diffraction intensity of *β*‐Si_3_N_4_ of the former electrode decreases during lithiation, confirming that *β*‐Si_3_N_4_ is partially consumed and not completely inactive. However, the crystalline structure of *β*‐Si_3_N_4_ is preserved. Even after 300 cycles, as shown in the high‐resolution TEM image in Figure S12, Supporting Information, the *β*‐Si_3_N_4_ crystals can be clearly observed. The *β*‐Si_3_N_4_ nanoparticles seem to be embedded in the matrix of amorphous Si and SEI. Also shown in Figure 7c is the high reversibility of Li_15_Si_4_ formation and dissolution upon cycling. The Si peak intensity decreases at the first lithiation but becomes more stable at the second lithiation. The introduction of *β*‐Si_3_N_4_ seems to stabilize the Si phase and thus alleviates the strong Li^+^ uptake reactions. Therefore, the electrode cyclability is improved. In contrast, as shown in Figure 7d (for the C—Si electrode), the Si intensity continuously and substantially fades upon cycling. Moreover, the Li_15_Si_4_ intensity at the second cycle is considerably higher than that at the first cycle, indicating that the Li^+^ uptake reactions, and thus the material volume change, were aggravated.

The crystal size (*L*) of the Si, *β*‐Si_3_N_4_, and Li_15_Si_4_ phases at various states of charge can be estimated using Scherrer's formula:^[^ ^61^ ^]^

(1) $$L=\frac{K\times \lambda }{B\;\cos\theta \;}$$

where *K* is the Scherrer constant, which can be considered to be 0.94 for spherical crystallites, *λ* is the X‐ray wavelength, and *B* is the full width at half maximum of the XRD peak at a diffraction angle of 2*θ*. The calculation results are shown in Figure 7e,f. The *β*‐Si_3_N_4_ crystal size slightly decreases during lithiation, confirming that the crystal is indeed consumed. With the incorporation of *β*‐Si_3_N_4_, the crystal size of Li_15_Si_4_ markedly decreases and the size variation upon lithiation and delithiation becomes more reversible compared to that of the C—Si electrode. It has been reported that a smaller Li_15_Si_4_ crystal size leads to improved electrode cycle life.^[^ ^62^, ^63^ ^]^ The crystal size of Si was found to be decreased when the *β*‐Si_3_N_4_ was introduced. However, as shown, the Si crystal size reduction upon cycling was somewhat mitigated. For the C—Si electrode, the Si crystal size significantly decreased and became nearly amorphous after two cycles. The operando XRD data confirm that the incorporated *β*‐Si_3_N_4_ indeed alters the phase evolution behavior of the electrode during charging and discharging.

**Figure 8:**
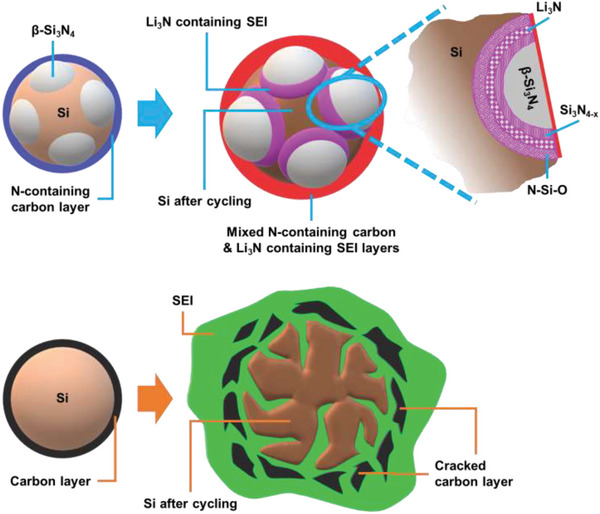
Schematic illustration of structure evolution in C‐*β*‐Si_3_N_4_/50%Si and C—Si electrodes after charge–discharge cycling.

**Figure** **8** shows the structure evolutions of the C‐*β*‐Si_3_N_4_/50%Si and C—Si electrodes. The former sample is coated by an N‐containing carbon layer (confirmed by the XPS pyrrolic nitrogen and C—N—O signals). The N doping increases the electronic conductivity and electrolyte affinity of the carbon layer.^[^ ^64^ ^]^ This is crucial for compensating for the low electronic conductivity of *β*‐Si_3_N_4_, which is partially consumed via the following conversion reaction:

(2) $$\beta {\mathrm{Si}}_{3}N_{4}+3x{\mathrm{Li}}^{+}+3xe^{-}⇄\beta {\mathrm{Si}}_{3}N_{4-x}+x{\mathrm{Li}}_{3}N$$

This reaction could be partially reversible. Besides, some shallow Li^+^ intercalation reactions and double‐layer charging reactions could take place, contributing to the measured capacity of *β*‐Si_3_N_4_. Upon cycling, *β*‐Si_3_N_4_ helps mechanically stabilize the electrode structure. As shown in the enlarged image, even though a Si_3_N_4‐_ *_x_* species formed, the crystalline structure of hexagonal *β*‐Si_3_N_4_ was preserved, alleviating the electrode volume variation. The formed Li_3_N at the *β*‐Si_3_N_4_/Si interface establishes a percolating Li^+^ conducting network across the composite electrode. In addition, due to the high robustness and good passivation ability of Li_3_N, the surface SEI layer is thin and stable. It is believed that there is a distorted Si layer (based on Raman data) with N and O content (based on XPS data) near the *β*‐Si_3_N_4_/Si interface that has relatively low activity toward Li^+^ uptake (i.e., low volume expansion). This layer can thus be a buffer intermediate that accommodates the strain generated at the interface, increasing electrode cyclability. In contrast, a conventional C—Si electrode has large volume variation during cycling. Thus, cracks easily form and particle pulverization occurs. The repeated breakdown and formation of the SEI lead to its continuous accumulation, which increases the Li^+^ transport distance and hinders the charge transfer reactions. Moreover, the thickened SEI gradually isolates the loosely connected Si and carbon fragments, causing rapid capacity deterioration.

**Figure 9:**
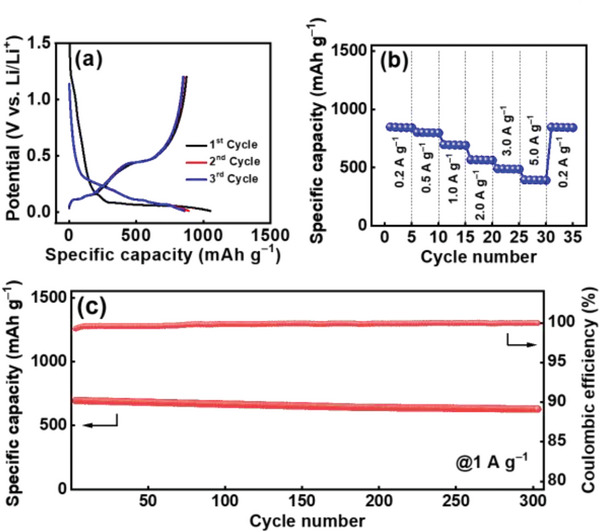
a) Initial three charge–discharge cycles, b) rate performance, and c) cycling stability of C‐*β*‐Si_3_N_4_/50%Si@AG electrode.

Next, AG was incorporated into C‐*β*‐Si_3_N_4_/50%Si with a weight ratio of 1:1. The corresponding charge–discharge profiles are shown in **Figure** **9**. The C‐*β*‐Si_3_N_4_/50%Si@AG electrode has reversible capacities of 849, 697, and 396 mAh g^−1^ at 0.2, 1, and 5 A g^−1^, respectively. Figure 9c shows the cycling stability data of the C‐*β*‐Si_3_N_4_/50%Si@AG electrode measured at 1 A g^−1^. The steady CE value reached >99.9%. After 300 charge–discharge cycles, the electrode retained a capacity of 628 mAh g^−1^, corresponding to 90% of the initial capacity. As shown in Table S5, Supporting Information, this is among the best electrode durability values reported in the literature. C‐*β*‐Si_3_N_4_/50%Si||NMC‐811 and C‐*β*‐Si_3_N_4_/50%Si@AG||NMC‐811 cells were assembled for the evaluation of full‐cell properties. Figure S13, Supporting Information, shows the obtained charge–discharge curves of the cells measured at various C rates. The gravimetric energy densities of the two cells based on the total mass of negative and positive electrode active materials are estimated to be ≈575 and ≈560 Wh kg^−1^, respectively, at 0.1 C. This reveals the merits of the proposed C‐*β*‐Si_3_N_4_/Si‐based composite anodes.

## 3 Conclusions

The superior lithiation/delithiation properties of *β*‐Si_3_N_4_ over *α*‐Si_3_N_4_ were confirmed. *β*‐Si_3_N_4_/Si composites were synthesized via a facile ball‐milling method and a simple wet chemical protocol was used to form an N‐containing carbon coating, where the nitrogen originated from *β*‐Si_3_N_4_. In contrast to the traditional understanding of crystalline Si_3_N_4_, which was thought to be electrochemically inactive, we found that the incorporated *β*‐Si_3_N_4_ substantially alters the charge–discharge performance of Si electrodes. The strongest synergistic effects of combining *β*‐Si_3_N_4_ and Si phases on the specific capacities were found for the C‐*β*‐Si_3_N_4_/50%Si electrode. Of note, the N‐containing carbon‐coated *β*‐Si_3_N_4_ moderately participated in the lithiation reactions. *β*‐Si_3_N_4_ was partially consumed (leaving behind Si_3_N_4‐_ *_x_*) to form Li_3_N, which is protective and highly Li^+^ conductive, both on the electrode surface and at the *β*‐Si_3_N_4_/Si interface. However, the crystalline structure of the hexagonal *β*‐Si_3_N_4_ was well preserved even after prolonged cycling, which mechanically stabilized the electrode, prevented Si particle agglomeration, and thus suppressed capacity degradation. Operando XRD results confirmed that with the incorporation of *β*‐Si_3_N_4_, the crystal size of Li_15_Si_4_ considerably decreased and the Li_15_Si_4_ formation and dissolution upon lithiation/delithiation became more reversible. Moreover, there is a distorted Si layer with N and O content near the *β*‐Si_3_N_4_/Si interface that has relatively low activity toward Li^+^ uptake (i.e., low volume expansion). This layer can be an intermediate to buffer the volume variation of the bulk Si phase, increasing electrode cyclability. The C‐*β*‐Si_3_N_4_/50%Si@AG electrode showed a reversible capacity of 849 mAh g^−1^ at 0.2 A g^−1^. After 300 cycles, this electrode retained 90% of its initial capacity with a steady CE value of above 99.9%. In addition, the C‐*β*‐Si_3_N_4_/50%Si||NMC‐811 and C‐*β*‐Si_3_N_4_/50%Si@AG||NMC‐811 full cells showed promising energy densities of ≈575 and ≈560 Wh kg^−1^ (based on active materials), respectively. The unique role of *β*‐Si_3_N_4_ in the enhancement of the electrochemical performance of Si‐based anodes was examined. The great potential for high‐energy‐density and high‐reliability LIB applications is expected.

## 4 Experimental Section

### Synthesis of Carbon‐Coated *α*‐Si_3_N_4_, *β*‐Si_3_N_4_, and *β*‐Si_3_N_4_/Si Composites

Commercial Si powder, *α*‐Si_3_N_4_ powder, and *β*‐Si_3_N_4_ powder were provided by Super Energy Material Inc., Taiwan. AG (*D*_50_: ≈16 µm, purity: >99.9%) was purchased from Shanghai Shanshan Technology Co. Ltd. *β*‐Si_3_N_4_/Si composites with various *β*‐Si_3_N_4_‐to‐Si ratios were prepared via high‐energy ball milling. Briefly, *β*‐Si_3_N_4_ and Si powders at various weight ratios (*β*‐Si_3_N_4_/Si = 25/75, 50/50, 75/25) were dispersed in anhydrous ethanol and transferred to a zirconia vessel for planetary ball milling (a powder/zirconia ball weight ratio of 1/10 was used). A carbon coating was applied to various powders using a wet chemical method.^[^ ^65^ ^]^ In this process, glucose was used as the carbon precursor and the carbonization was performed at 850 °C under Ar for 5 h.

### Cell Assembly

The anode active material, Super P, and sodium polyacrylate binder were mixed in a 7:2:1 ratio with deionized water. The slurry was cast onto Cu foil using a doctor blade and then vacuum‐dried at 100 °C for 6 h. The obtained electrode was punched to match the required dimensions of a CR2032 coin cell. The active material mass loading was ≈2 mg cm^−2^. Li foil and a glass fiber membrane were used as the counter electrode and separator, respectively. For the full cell construction, the developed anode was paired with a LiNi_0.8_Mn_0.1_Co_0.1_O_2_ (NMC‐811) cathode with an anode‐to‐cathode capacity ratio of 1.15:1. The anode was pre‐conditioned for two cycles in half cells prior to the full cell assembly. An electrolyte composed of 1 m LiPF_6_ salt, ethylene carbonate/diethyl carbonate mixed solvent (1:1 by volume), and 5 wt.% fluoroethylene carbonate additive was adopted. The coin cells were assembled in an Ar‐filled glove box (Vigor Tech. Co. Ltd.), where both the moisture and oxygen content levels were maintained below 0.1 ppm.

### Material and Electrochemical Characterizations

The crystallinity of the samples was characterized by XRD (Bruker D2 Phaser). The phase composition was determined using a Partial Or No Known Crystal Structure method,^[^ ^66^ ^]^ which was implemented in the Bruker TOPAS software. The Raman spectra were collected using a LabRAM HR 800 spectrometer with an excitation laser wavelength of 633 nm. Thermogravimetric analysis (TGA; TA Instruments Q500) was conducted under air with a heating rate of 5 °C min^−1^. SEM (JEOL JCM‐7000 NeoScope), TEM (JEOL F200), and energy‐dispersive X‐ray spectroscopy (EDS) were used to examine the morphologies, microstructures, and chemical compositions of the samples, respectively. XPS (Thermo Fisher Scientific ESCALAB 250Xi) was used to analyze the surface chemical states. Monochromatic Al K_*α*_ radiation (1486.6 eV) was adopted as the X‐ray source. The particle size distribution of various samples was estimated using a DLS (Otsuka ELSZ‐2000) method, in which anhydrous ethanol was used to disperse the particles. Cyclic voltammetry (CV; BioLogic BCS‐810) was performed in the range of 0.01–2.0 V versus Li/Li^+^ with a potential sweep rate of 0.1 mV s^−1^. EIS analysis was conducted with a frequency range of 10^6^–10^−2^ Hz and an AC amplitude of 10 mV. The charge–discharge properties, in terms of capacity, rate capability, and cycling stability, of various electrodes were evaluated using a NEWARE CT‐4000 battery tester at 25 °C. For the operando XRD analyses, the cells were subjected to synchrotron X‐ray examination during charging/discharging at a rate of 0.5 A g^−1^, which was performed at Beamline TPS‐19A of the National Synchrotron Radiation Research Center in Taiwan.

### Statistical Analysis

The DLS, CV, EIS, GITT, and charge–discharge measurements of various electrodes were repeated at least three times to ensure validity. The performance deviation was typically within ≈5% and the reported data were the medians. All the XPS spectra were calibrated with the binding energy of C 1s peak at 284.7 eV. The data fitting was done using XPSPEAK 4.1 software. For XRD data, the background subtraction and phase identification were conducted using the EVA and TOPAS programs provided in Bruker software package. The Origin software was used for data analysis and processing.

## Conflict of Interest

The authors declare no conflict of interest.

## Supporting information

Supporting Information

## Data Availability

The data that support the findings of this study are available from the corresponding author upon reasonable request.

## References

[1] J. B. Goodenough , K. S. Park , J. Am. Chem. Soc. 2013, 135, 1167. 23294028 10.1021/ja3091438

[2] P. Li , G. Q. Zhao , X. B. Zheng , X. Xu , C. H. Yao , W. P. Sun , S. X. Dou , Energy Storage Mater. 2018, 15, 422.

[3] J. R. Szczech , S. Jin , Energy Environ. Sci. 2011, 4, 56.

[4] M. Gu , Y. He , J. M. Zheng , C. M. Wang , Nano Energy 2015, 17, 366.

[5] M. N. Obrovac , V. L. Chevrier , Chem. Rev. 2014, 114, 11444. 25399614 10.1021/cr500207g

[6] X. H. Liu , J. Y. Huang , Energy Environ. Sci. 2011, 4, 3844.

[7] J. Lu , Z. W. Chen , F. Pan , Y. Cui , K. Amine , Electrochem. Energy Rev. 2018, 1, 35.

[8] R. F. H. Hernandha , P. C. Rath , B. Umesh , J. Patra , C. Y. Huang , W. W. Wu , Q. F. Dong , J. Li , J. K. Chang , Adv. Funct. Mater. 2021, 31, 2104135.

[9] N. Liu , Z. Lu , J. Zhao , M. T. McDowell , H. W. Lee , W. Zhao , Y. Cui , Nat. Nanotechnol. 2014, 9, 187. 24531496 10.1038/nnano.2014.6

[10] M. Ashuri , Q. R. He , Y. Z. Liu , K. Zhang , S. Emani , M. S. Sawicki , J. S. Shamie , L. L. Shaw , Electrochim. Acta 2016, 215, 126.

[11] H. Jia , X. Li , J. Song , X. Zhang , L. Luo , Y. He , B. Li , Y. Cai , S. Hu , X. Xiao , C. Wang , K. M. Rosso , R. Yi , R. Patel , J. G. Zhang , Nat. Commun. 2020, 11, 1474. 32193387 10.1038/s41467-020-15217-9 PMC7081208

[12] Y. Yang , W. Yuan , W. Q. Kang , Y. T. Ye , Q. Q. Pan , X. Q. Zhang , Y. Z. Ke , C. Wang , Z. Q. Qiu , Y. Tang , Sustainable Energy Fuels 2020, 4, 1577.

[13] W. Wang , L. Gu , H. L. Qian , M. Zhao , X. Ding , X. S. Peng , J. Sha , Y. W. Wang , J. Power Sources 2016, 307, 410.

[14] X. Y. Zhang , W. L. Song , Z. L. Liu , H. S. Chen , T. Li , Y. J. Wei , D. N. Fang , J. Mater. Chem. A 2017, 5, 12793.

[15] L. Sun , Y. X. Liu , R. Shao , J. Wu , R. Y. Jiang , Z. Jin , Energy Storage Mater. 2022, 46, 482.

[16] H. Kim , E. J. Lee , Y. K. Sun , Mater. Today 2014, 17, 285.

[17] H. C. Song , H. X. Wang , Z. X. Lin , X. F. Jiang , L. W. Yu , J. Xu , Z. W. Yu , X. W. Zhang , Y. J. Liu , P. He , L. J. Pan , Y. Shi , H. S. Zhou , K. J. Chen , Adv. Funct. Mater. 2016, 26, 524.

[18] Y. Chen , J. Qian , Y. Cao , H. Yang , X. Ai , ACS Appl. Mater. Interfaces 2012, 4, 3753. 22757774 10.1021/am300952b

[19] P. X. Zhang , L. Huang , Y. L. Li , X. Z. Ren , L. B. Deng , Q. H. Yuan , Electrochim. Acta 2016, 192, 385.

[20] K. M. Lee , Y. S. Lee , Y. W. Kim , Y. K. Sun , S. M. Lee , J. Alloys Compd. 2009, 472, 461.

[21] S. Chae , M. Ko , S. Park , N. Kim , J. Ma , J. Cho , Energy Environ. Sci. 2016, 9, 1251.

[22] H. I. Park , M. Sohn , J. H. Choi , C. Park , J. H. Kim , H. Kim , Electrochim. Acta 2016, 210, 301.

[23] S. B. Son , S. C. Kim , C. S. Kang , T. A. Yersak , Y. C. Kim , C. G. Lee , S. H. Moon , J. S. Cho , J. T. Moon , K. H. Oh , S. H. Lee , Adv. Energy Mater. 2012, 2, 1226.

[24] Y. Domi , H. Usui , R. Takaishi , H. Sakaguchi , ChemElectroChem 2019, 6, 581.

[25] R. A. Vila , W. Huang , Y. Cui , Cell Rep. Phys. Sci. 2020, 1, 100188.

[26] C. Zhan , T. P. Wu , J. Lu , K. Amine , Energy Environ. Sci. 2018, 11, 243.

[27] J. S. Edge , S. O'Kane , R. Prosser , N. D. Kirkaldy , A. N. Patel , A. Hales , A. Ghosh , W. Ai , J. Chen , J. Yang , S. Li , M. C. Pang , L. Bravo Diaz , A. Tomaszewska , M. W. Marzook , K. N. Radhakrishnan , H. Wang , Y. Patel , B. Wu , G. J. Offer , Phys. Chem. Chem. Phys. 2021, 23, 8200. 33875989 10.1039/d1cp00359c

[28] O. C. Harris , Y. X. Lin , Y. Qi , K. Leung , M. H. Tang , J. Electrochem. Soc. 2020, 167, 013502.

[29] X. N. Zhang , G. L. Pan , G. R. Li , J. Q. Qu , X. P. Gao , Solid State Ionics 2007, 178, 1107.

[30] S. J. Kim , M. C. Kim , S. B. Han , G. H. Lee , H. S. Choe , D. H. Kwak , S. Y. Choi , B. G. Son , M. S. Shin , K. W. Park , Nano Energy 2016, 27, 545.

[31] Z. X. Xiao , C. Lei , C. H. Yu , X. Chen , Z. X. Zhu , H. R. Jiang , F. Wei , Energy Storage Mater. 2020, 24, 565.

[32] X. L. Qu , X. Zhang , Y. J. Wu , J. J. Hu , M. X. Gao , H. G. Pan , Y. F. Liu , J. Power Sources 2019, 443, 227265.

[33] F. L. Riley , J. Am. Ceram. Soc. 2000, 83, 245.

[34] J. F. Yang , T. Ohji , S. Kanzaki , A. Diaz , S. Hampshire , J. Am. Ceram. Soc. 2002, 85, 1512.

[35] E. T. Turkdogan , P. M. Bills , V. A. Tippett , J. Appl. Chem. 1958, 8, 296.

[36] G. Ziegler , J. Heinrich , G. Wotting , J. Mater. Sci. 1987, 22, 3041.

[37] R. Grün , Acta Crystallogr., Sect. B: Struct. Sci., Cryst. Eng. Mater. 1979, 35, 800.

[38] R. J. Lad , in Physical Structure, (Ed: W. N. Unertl ), Elsevier Science B. V, North‐Holland 1996, Vol. 1, Ch. 5.

[39] A. C. Ferrari , J. Robertson , Phys. Rev. B 2000, 61, 14095.

[40] H. J. Scheibe , D. Drescher , P. Alers , Fresenius' J. Anal. Chem. 1995, 353, 695.

[41] A. E. Kaloyeros , F. A. Jové , J. Goff , B. Arkles , ECS J. Solid State Sci. Technol. 2017, 6, P691.

[42] K. Kijima , S. Shirasaki , J. Chem. Phys. 1976, 65, 2668.

[43] J. D. Xie , H. Y. Li , B. Umesh , T. C. Lee , J. K. Chang , Y. A. Gandomi , Electrochim. Acta 2018, 292, 951.

[44] J. Patra , P. C. Rath , C. Li , H. M. Kao , F. M. Wang , J. Li , J. K. Chang , ChemSusChem 2018, 11, 3923. 30251351 10.1002/cssc.201801962

[45] P. Lengsfeld , S. Brehme , K. Brendel , C. Genzel , N. H. Nickel , Phys. Status Solidi B 2003, 235, 170.

[46] D. C. Lin , Z. D. Lu , P. C. Hsu , H. R. Lee , N. Liu , J. Zhao , H. T. Wang , C. Liu , Y. Cui , Energy Environ. Sci. 2015, 8, 2371.

[47] G. Ge , G. Li , X. Wang , X. Chen , L. Fu , X. Liu , E. Mao , J. Liu , X. Yang , C. Qian , Y. Sun , Nano Lett. 2021, 21, 3127. 33734706 10.1021/acs.nanolett.1c00317

[48] C. Zhang , F. Wang , J. Han , S. Bai , J. Tan , J. Liu , F. Li , Small Struct. 2021, 2, 2100009.

[49] Y. Surace , D. Leanza , M. Mirolo , Ł. Kondracki , C. A. F. Vaz , M. El Kazzi , P. Novák , S. Trabesinger , Energy Storage Mater. 2022, 44, 156.

[50] I. A. Profatilova , C. Stock , A. Schmitz , S. Passerini , M. Winter , J. Power Sources 2013, 222, 140.

[51] M. N. Obrovac , L. J. Krause , J. Electrochem. Soc. 2007, 154, A103.

[52] Q. Wang , M. Zhu , G. Chen , N. Dudko , Y. Li , H. Liu , L. Shi , G. Wu , D. Zhang , Adv. Mater. 2022, 34, 2109658. 10.1002/adma.202109658 35172027

[53] C. H. Jung , K. H. Kim , S. H. Hong , ACS Appl. Mater. Interfaces 2019, 11, 26753. 31276371 10.1021/acsami.9b03866

[54] K. Pan , F. Zou , M. Canova , Y. Zhu , J. H. Kim , J. Power Sources 2019, 413, 20.

[55] B. Umesh , P. C. Rath , J. Patra , R. F. H. Hernandha , S. B. Majumder , X. Gao , D. Bresser , S. Passerini , H. Z. Lai , T. L. Chang , J. K. Chang , Chem. Eng. J. 2022, 430, 132693.

[56] J. H. Yang , R. C. de Guzman , S. O. Salley , K. Y. S. Ng , B. H. Chen , M. M. C. Cheng , J. Power Sources 2014, 269, 520.

[57] S. X. Mei , S. G. Guo , B. Xiang , J. G. Deng , J. J. Fu , X. M. Zhang , Y. Zheng , B. Gao , P. K. Chu , K. F. Huo , J. Energy Chem. 2022, 69, 616.

[58] Y. M. Sun , Y. B. Li , J. Sun , Y. Z. Li , A. Pei , Y. Cui , Energy Storage Mater. 2017, 6, 119.

[59] S. Chae , S. Park , K. Ahn , G. Nam , T. Lee , J. Sung , N. Kim , J. Cho , Energy Environ. Sci. 2020, 13, 1212.

[60] U. v. Alpen , J. Solid State Chem. 1979, 29, 379.

[61] H. P. Klug , L. E. Alexander , X‐Ray Diffraction Procedures: For Polycrystalline and Amorphous Materials, John Wiley & Sons, Inc., New York, USA 1974.

[62] Y. Domi , H. Usui , K. Sugimoto , H. Sakaguchi , Energy Technol. 2019, 7, 1800946.

[63] D. S. M. Iaboni , M. N. Obrovac , J. Electrochem. Soc. 2015, 163, A255.

[64] Sutarsis , J. Patra , C. Y. Su , J. Li , D. Bresser , S. Passerini , J. K. Chang , ACS Appl. Mater. Interfaces 2020, 12, 32797. 32559066 10.1021/acsami.0c08440

[65] B. Umesh , P. C. Rath , R. F. H. Hernandha , J. Y. Lin , S. B. Majumder , Q. F. Dong , J. K. Chang , ACS Sustainable Chem. Eng. 2020, 8, 16252.

[66] N. V. Y. Scarlett , I. C. Madsen , Powder Diffr. 2006, 21, 278.

