# Supplementary material for: N‐Containing Carbon‐Coated β‐Si3N4 Enhances Si Anodes for High‐Performance Li‐Ion Batteries
Source: Adv Sci (Weinh). 2023 May 11;10(21):2301218. doi: 10.1002/advs.202301218 (PMC10375156; doi:10.1002/advs.202301218)
Supplement: Supplementary file 1 — Supporting Information [file ADVS-10-2301218-s001.pdf]

ADVANCED SCIENCE
Open Access

Supporting Information

for *Adv. Sci.*, DOI 10.1002/advs.202301218

N-Containing Carbon-Coated β-Si_3N_4 Enhances Si Anodes for High-Performance Li-Ion Batteries

*Rahmandhika Firdauzha Hary Hernandha, Bharath Umesh, Purna Chandra Rath, Le Thi Thu Trang, Ju-Chao Wei, Yu-Chun Chuang, Ju Li and Jeng-Kuei Chang**

Supporting Information

N-containing carbon-coated β-Si_3N_4 enhances Si anodes for high-performance Li-ion batteries

*Rahmandhika Firdauzha Hary Hernandha, Bharath Umesh, Purna Chandra Rath, Le Thi Thu Trang, Ju-Chao Wei, Yu-Chun Chuang, Ju Li, Jeng-Kuei Chang**

Table S1. Electronic conductivity data of various samples.

Samples	Electronic conductivity ($\times 10^{-1}$ S cm^{-1})
C-α-Si_3N_4	1.02
C-β-Si_3N_4	1.07
C-β-Si_3N_4/25%Si	1.51
C-β-Si_3N_4/50%Si	1.92
C-β-Si_3N_4/75%Si	2.35
C-Si	2.76

Table S2. Reversible specific capacities of α-Si_3N_4, β-Si_3N_4, C-α-Si_3N_4, and C-β-Si_3N_4 electrodes measured at various specific currents.

Current rate (A g^{-1})	α-Si_3N_4 (mAh g^{-1})	β-Si_3N_4 (mAh g^{-1})	C-α-Si_3N_4 (mAh g^{-1})	C-β-Si_3N_4 (mAh g^{-1})
0.05	80	91	109	118
0.1	71	86	100	106
0.3	58	67	84	92
0.5	51	61	76	83
1	42	52	64	73
2	31	42	52	62
High rate retention*	39%	46%	48%	53%

* a comparison between reversible capacities at 2 and 0.05 A g^{-1}.

Table S3. Tap densities of various samples.

Samples	Tap density (g cm^{-3})
C-β-Si_3N_4	0.887
C-β-Si_3N_4/25%Si	0.819
C-β-Si_3N_4/50%Si	0.802
C-β-Si_3N_4/75%Si	0.797
C-Si	0.572

Table S4. Expected capacities of various C-β-Si_3N_4/Si composite anodes calculated from linear combination of measured capacities of C-β-Si_3N_4 and C-Si electrodes. Synergistic effects are shown.

Current rate (A g^{-1})	C-β-Si_3N_4 (mAh g^{-1})	C-β-Si_3N_4/25%Si (mAh g^{-1})		$\frac{(A)}{(E)}$	C-β-Si_3N_4/50%Si (mAh g^{-1})		$\frac{(A)}{(E)}$	C-β-Si_3N_4/75%Si (mAh g^{-1})		$\frac{(A)}{(E)}$	C-Si (mAh g^{-1})
		Actual capacity (A)	Expected capacity (E)		Actual capacity (A)	Expected capacity (E)		Actual capacity (A)	Expected capacity (E)		
0.2	100	583	530	1.10	1068	960	1.11	1390	1390	1.00	1820
0.5	83	525	460	1.14	1000	837	1.19	1218	1214	1.00	1591
1	73	476	382	1.24	935	692	1.35	1124	1002	1.12	1311
2	62	424	297	1.43	769	532	1.45	930	766	1.21	1001
3	55	365	232	1.57	670	410	1.63	728	588	1.24	765
5	46	250	144	1.74	488	242	2.02	452	339	1.33	437

Table S5. Performance comparison of various Si-based composite anodes reported in the literature.

No.	Material	Synthesis method	Initial reversible capacity (mAh g^{-1})	Initial Coulombic efficiency (%)	Capacity retention (%) / number of cycles / current rate (A g^{-1})	References
1	α-Si_3N_4	Ball milling (reduce size with time dependent)	83	~40	N/A	[1]
2	α-Si_3N_4w + 30 % Si composites	Ball milling	>800	~40	~27 / 50 / 0.08	[2]
3	a-Si_3N_4p + 30 % Si composites	Ball milling	~1000	~50	~47 / 50 / 0.08	[2]
4	$Si_{3-x}M_xN_4$ (M=Fe)	Manual mix of α-Si_3N_4+Fe and pelletized	470.6	50.5	45.4 / 50 / 0.1 C	[3]
5	$Ti_{28}Si_{72}$ melt-spun powders	Ball milling of pre-alloyed melt-spun Ti-Si	~260	~67	~85 / ~47 / 2 x 10^{-4} A cm^{-2}	[4]
6	Binder-free a-SiN/BCNT	Hot filament CVD & sputtering	607	N/A	62 / 10 / 2 x 10^{-4} A cm^{-2}	[5]
7	Cu-coated Si (empowered by CuO, Cu_3Si, SiO, and SiO_2) nanowires	CVD and magnetron sputtering	2679	90.3	59 / 50 / 0.5 C	[6]
8	Si-Ti-Ni ternary nanoalloy	Single-roller melt-spinning technique	1158	87.7	~82 / 50 / 8.8 x 10^{-4} A cm^{-2}	[7]
9	$FeSi_2$/Si@C Nanocomposite	Two-step ball-milling process	942	72	93 / 200 / 0.1	[8]
10	a-$SiN_{0.92}$	Pulsed Laser Deposition	1800	~75	72.2 / 100 / 0.2 C	[9]
11	a-SiN_x/graphene	CVD, dispersion, and heat treatment	>3000	70	45.2 / 200 / 0.5	[10]
12	Hollow silicon nanosphere	Sol-gel method, magnesiothermic and hydrogen reductions, and acid etching	1610	72.2	~80 / 120 / 1	[11]

13	C-coated Si nanotube	Two-step CVD and selective thermal reduction etching	~3823	87	~81 / 80 (20th–100th) / 1 C	[12]
14	Si@α-Si_3N_4/CNF	Mixing & electrospinning	613	61.4	55.5 / 2000 / 10	[13]
15	N1-Si/CMAs (a-$SiN_{0.73}$)	Two-step DC sputtering on Cu Micro-cone arrays (CMAs)	2789	80	40 / 200 / 0.2 C	[14]
16	Si/$TiFeSi_2$ Nanocomposite	Melt spinning fabrication method and ball-milling	~1000	~80	~90 / 100 / 0.2	[15]
17	Si–Cu alloy (mixture) nanotubes	Vapor-solid oxidation mechanism, plasma enhanced CVD, and heat treatment	~1700	42	76.2 / 100 / 3.2	[16]
18	Si/Ni_3Si-Encapulated CNF Composites	One-pot electrospinning method	~855	72.5	N/A (capacity is increasing during cycling)	[17]
19	Micron-sized Fe–Cu–Si ternary composite	CVD, spray-drying, and elevated temperature reduction process	1287	91.4	90 / 50 / 0.65	[18]
20	a-SiN_x-H	Plasma Enhanced CVD	32	69.1	N/A (capacity was increasing until 200th and lowering until 350th cycle)	[19]
21	Si@Si_3N_4@C	Two-step gas–solid reaction	3093.8	91.5	81.3 / 200 / 0.5	[20]
22	$SiN_{0.7}$ nanoparticles	Gas-phase synthesis	1212	~55	82 / 300 / 0.5 C	[21]
23	p-Si@SiN	Direct nitriding hydrogenated porous Si	2584	81	84 / 200 / 0.5	[22]
**24**	**C-β-Si_3N_4/50%Si**	**Ball milling & wet chemical coating**	**1068**	**78**	**75 / 300 / 1**	**This work**
**25**	**C-β-Si_3N_4/50%Si@AG**		**849**	**83**	**90 / 300 / 1**	**This work**

References

[1] M. Martín-Gil, M. E. Rabanal, A. Várez, A. Kuhn, F. García-Alvarado, *Mater. Lett.* **2003**, *57*, 3063.

[2] X. N. Zhang, G. L. Pan, G. R. Li, J. Q. Qu, X. P. Gao, *Solid State Ion.* **2007**, *178*, 1107.

[3] N. Kalaiselvi, *Int. J. Electrochem. Sci.* **2007**, *2*, 478.

[4] K. M. Lee, Y. S. Lee, Y. W. Kim, Y. K. Sun, S. M. Lee, *J. Alloys Compd.* **2009**, *472*, 461.

[5] S. L. Katar, D. Hernandez, A. B. Labiosa, E. Mosquera-Vargas, L. Fonseca, B. Weiner, G. Morell, *Electrochim. Acta* **2010**, *55*, 2269.

[6] H. Chen, Y. Xiao, L. Wang, Y. Yang, *J. Power Sources* **2011**, *196*, 6657.

[7] S. B. Son, S. C. Kim, C. S. Kang, T. A. Yersak, Y. C. Kim, C. G. Lee, S. H. Moon, J. S. Cho, J. T. Moon, K. H. Oh, S. H. Lee, *Adv. Energy Mater.* **2012**, *2*, 1226.

[8] Y. Chen, J. Qian, Y. Cao, H. Yang, X. Ai, *ACS Appl. Mater. Interfaces* **2012**, *4*, 3753.

[9] N. Suzuki, R. B. Cervera, T. Ohnishi, K. Takada, *J. Power Sources* **2013**, *231*, 186.

[10] R. C. de Guzman, J. Yang, M. M. C. Cheng, S. O. Salley, K. Y. S. Ng, *J. Mater. Chem. A* **2014**, *2*, 14577.

[11] M. Ashuri, Q. R. He, Y. Z. Liu, K. Zhang, S. Emani, M. S. Sawicki, J. S. Shamie, L. L. Shaw, *Electrochim. Acta* **2016**, *215*, 126.

[12] W. Wang, L. Gu, H. L. Qian, M. Zhao, X. Ding, X. S. Peng, J. Sha, Y. W. Wang, *J. Power Sources* **2016**, *307*, 410.

[13] S. J. Kim, M. C. Kim, S. B. Han, G. H. Lee, H. S. Choe, D. H. Kwak, S. Y. Choi, B. G. Son, M. S. Shin, K. W. Park, *Nano Energy* **2016**, *27*, 545.

[14] C. Y. Wu, C. C. Chang, J. G. Duh, *J Power Sources* **2016**, *325*, 64.

[15] H. I. Park, M. Sohn, J. H. Choi, C. Park, J. H. Kim, H. Kim, *Electrochim. Acta* **2016**, *210*, 301.

[16] H. C. Song, H. X. Wang, Z. X. Lin, X. F. Jiang, L. W. Yu, J. Xu, Z. W. Yu, X. W. Zhang, Y. J. Liu, P. He, L. J. Pan, Y. Shi, H. S. Zhou, K. J. Chen, *Adv. Funct. Mater.* **2016**, *26*, 524.

[17] P. X. Zhang, L. Huang, Y. L. Li, X. Z. Ren, L. B. Deng, Q. H. Yuan, *Electrochim. Acta* **2016**, *192*, 385.

[18] S. Chae, M. Ko, S. Park, N. Kim, J. Ma, J. Cho, *Energy Environ. Sci.* **2016**, *9*, 1251.

[19] X. D. Huang, X. F. Gan, F. Zhang, Q. A. Huang, J. Z. Yang, *Electrochim. Acta* **2018**, *268*, 241.

[20] Z. X. Xiao, C. Lei, C. H. Yu, X. Chen, Z. X. Zhu, H. R. Jiang, F. Wei, *Energy Storage Mater.* **2020**, *24*, 565.

[21] S. O. Kilian, H. Wiggers, *Part. Part. Syst. Charact.* **2021**, *38*, 2100007.

[22] S. X. Mei, S. G. Guo, B. Xiang, J. G. Deng, J. J. Fu, X. M. Zhang, Y. Zheng, B. Gao, P. K. Chu, K. F. Huo, *J. Energy Chem.* **2022**, *69*, 616.

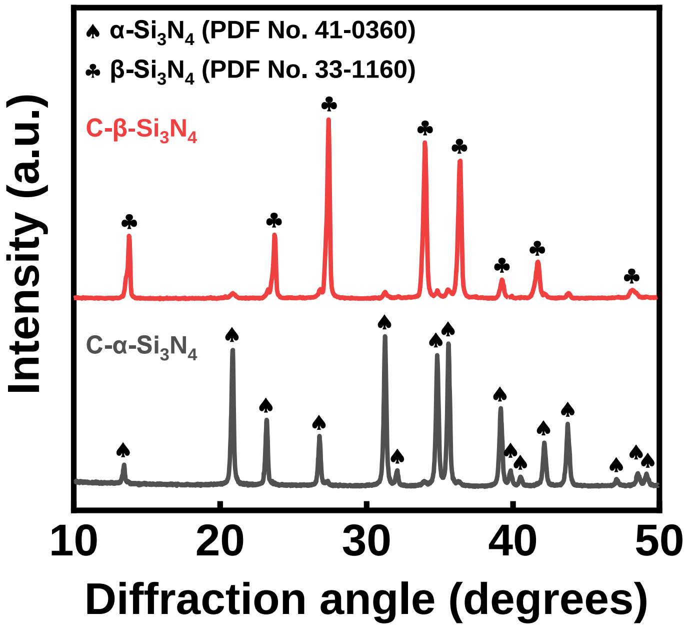

Figure S1. XRD patterns of C-α-Si_3N_4 and C-β-Si_3N_4.

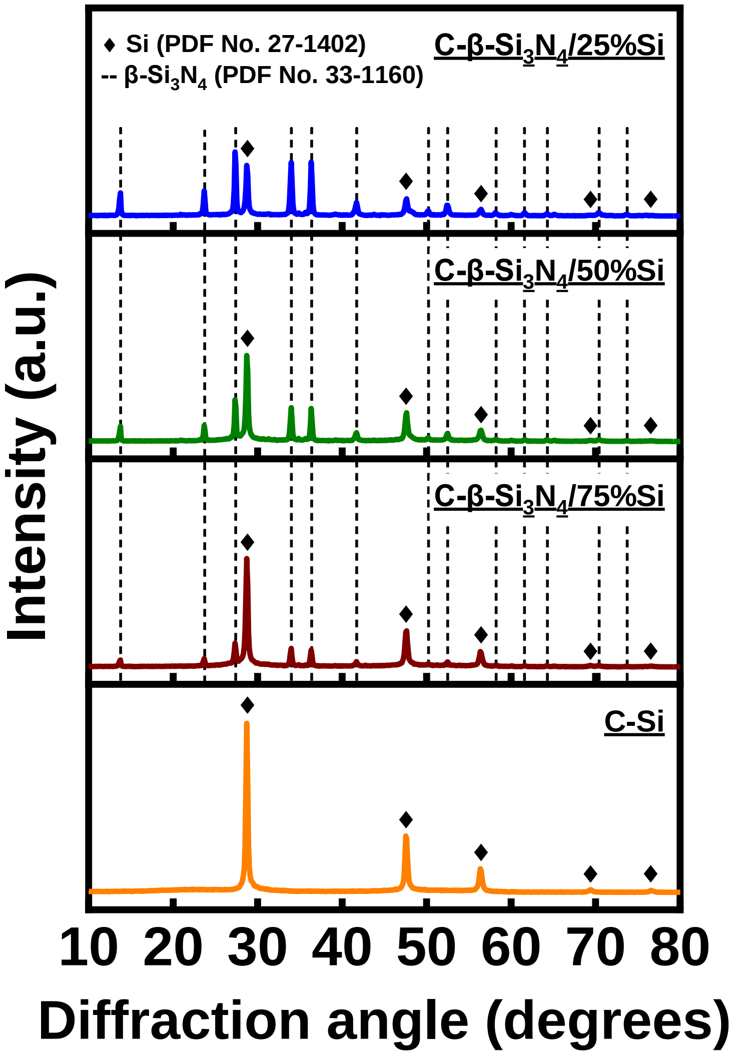

Figure S2. XRD patterns of C-β-Si_3N_4/25%Si, C-β-Si_3N_4/50%Si, C-β-Si_3N_4/75%Si, and C-Si samples.

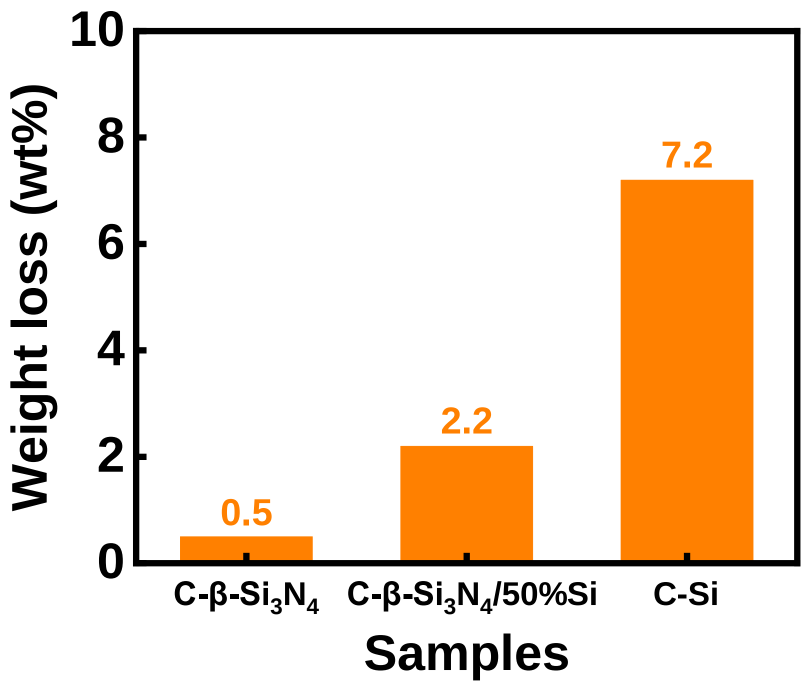

Figure S3. Weight loss data for C-β-Si_3N_4, C-β-Si_3N_4/50%Si, and C-Si after immersion in 25 mM HF aqueous solution at 25 °C for 1 h.

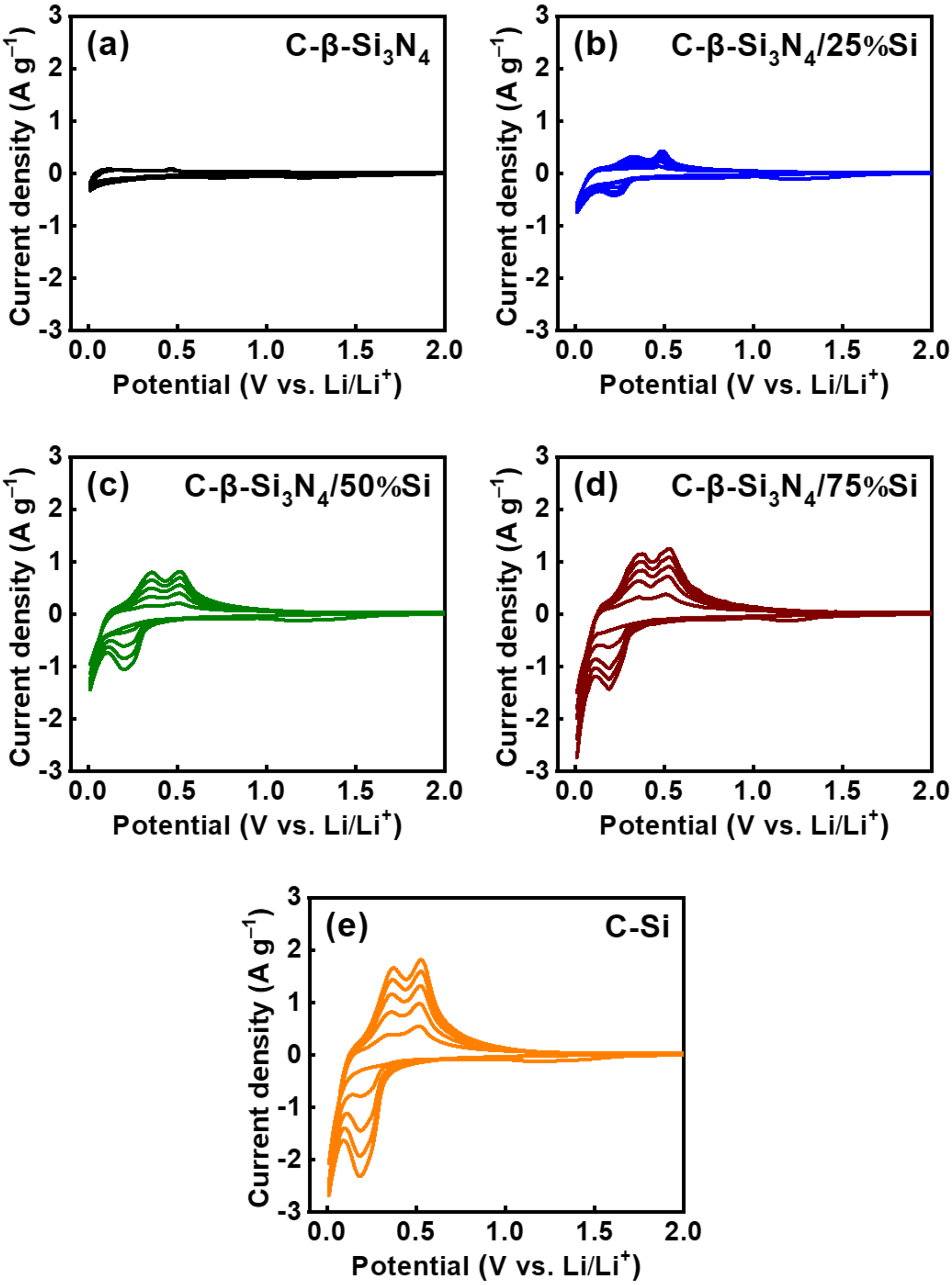

Figure S4. CV curves of (a) C-β-Si_3N_4, (b) C-β-Si_3N_4/25%Si, (c) C-β-Si_3N_4/50%Si, (d) C-β-Si_3N_4/75%Si, and (e) C-Si electrodes recorded with potential scan rate of 0.1 mV s^{-1} for 5 cycles.

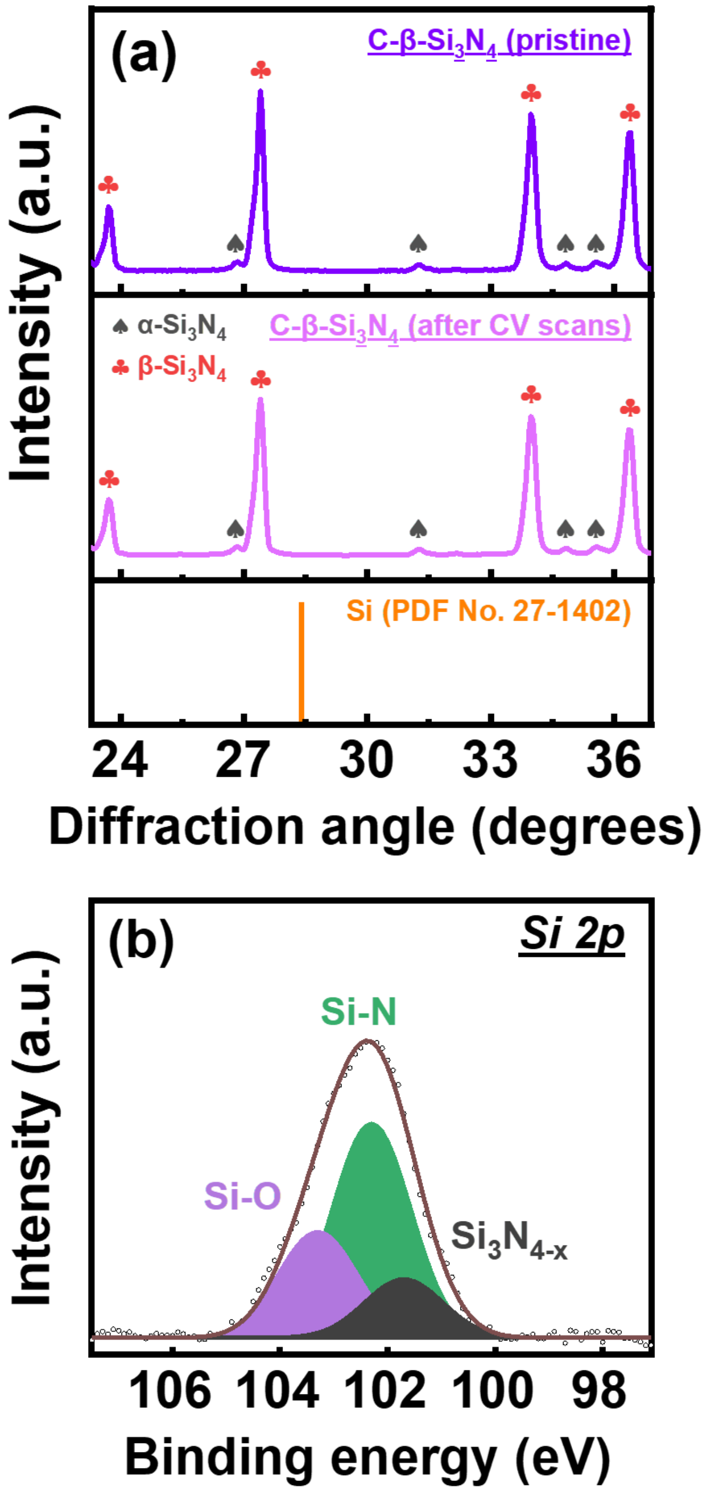

Figure S5. (a) XRD data of C-β-Si_3N_4 electrode before and after CV scans. (b) XPS Si 2p spectrum of C-β-Si_3N_4 electrode after CV scans.

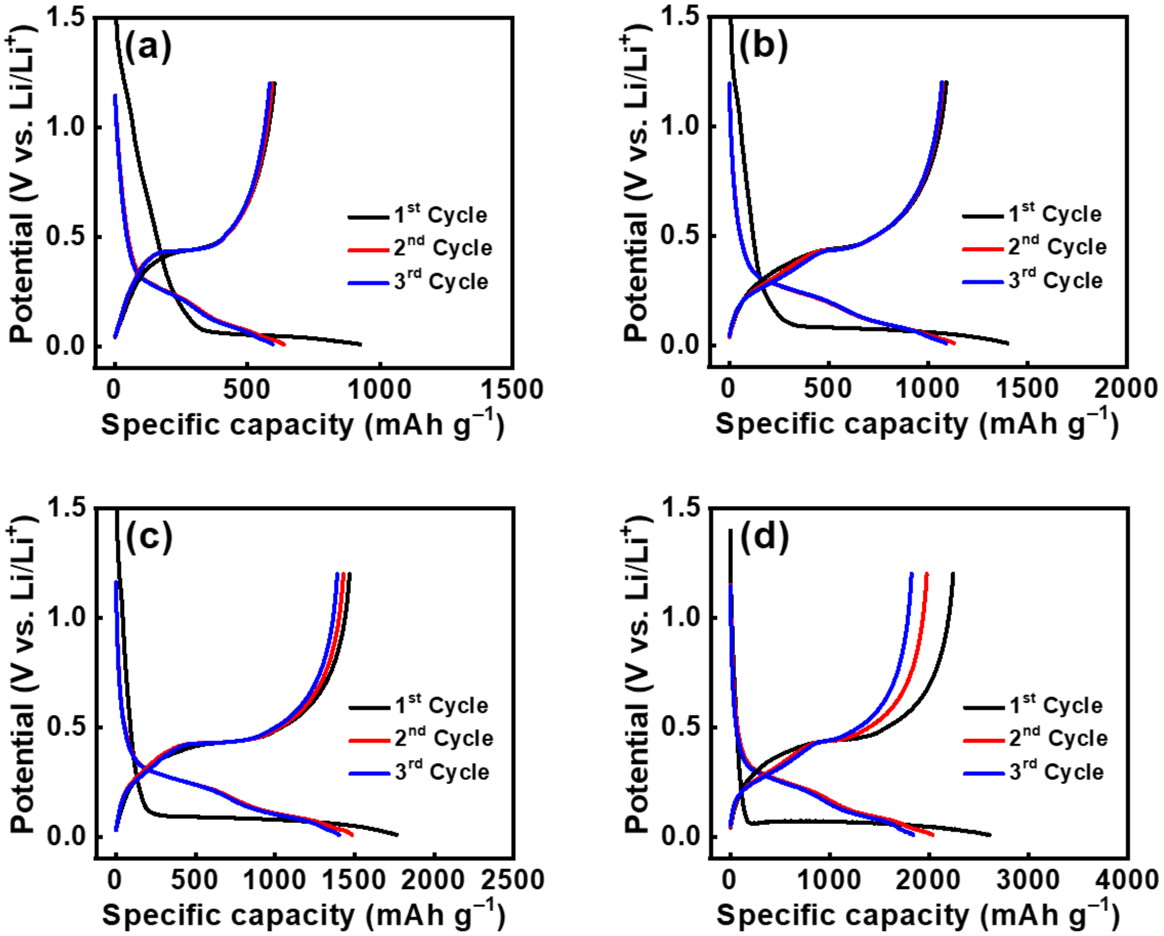

Figure S6. Initial three charge–discharge cycles of (a) C-β-Si_3N_4/25%Si, (b) C-β-Si_3N_4/50%Si, (c) C-β-Si_3N_4/75%Si, and (d) C-Si electrodes measured at 0.2 A g^{-1}.

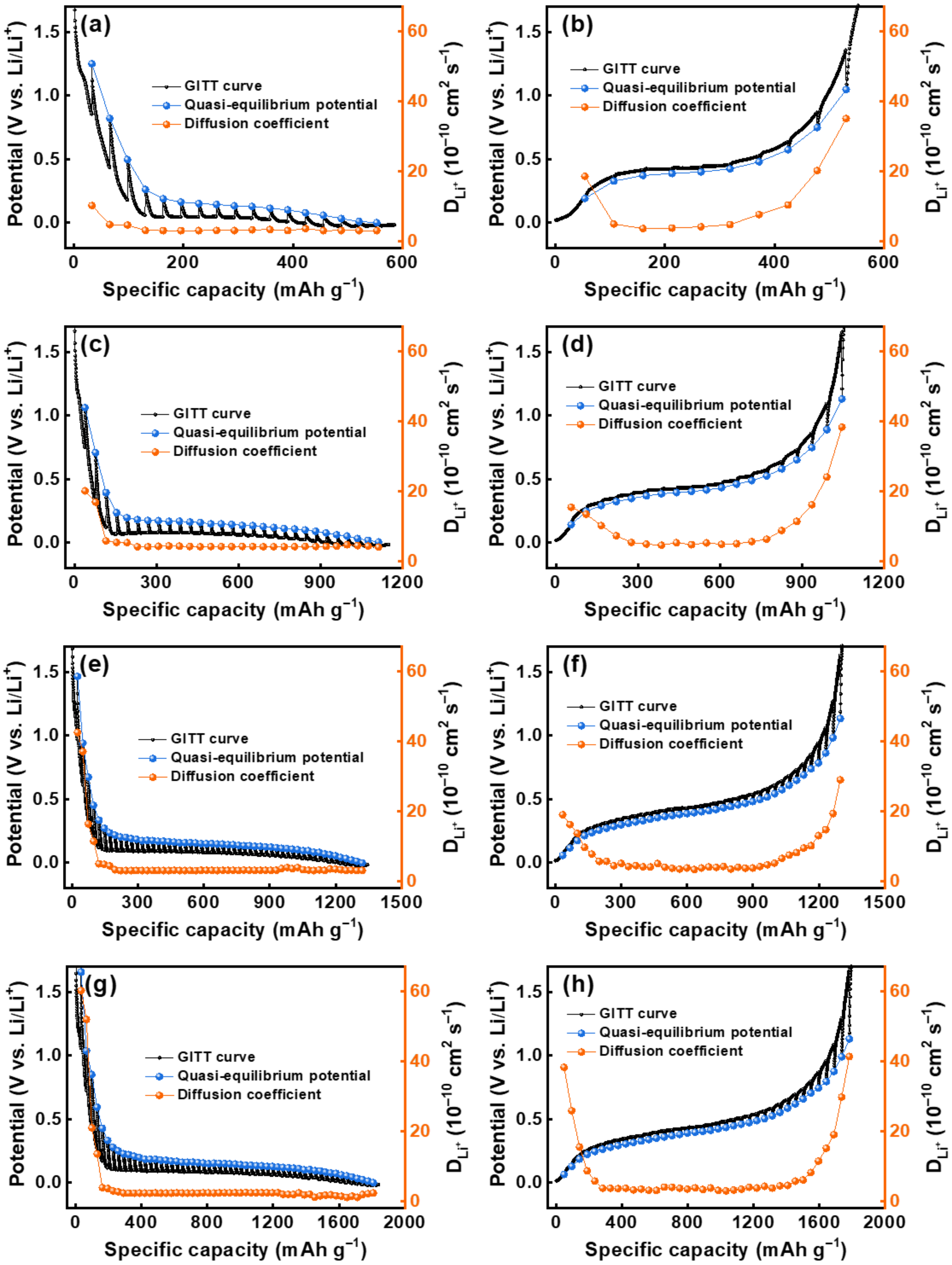

Figure S7. GITT measurements of (a, b) C-β-Si_3N_4/25%Si, (c, d) C-β-Si_3N_4/50%Si, (e, f) C-β-Si_3N_4/75%Si, and (g, h) C-Si electrodes during lithiation and delithiation processes.

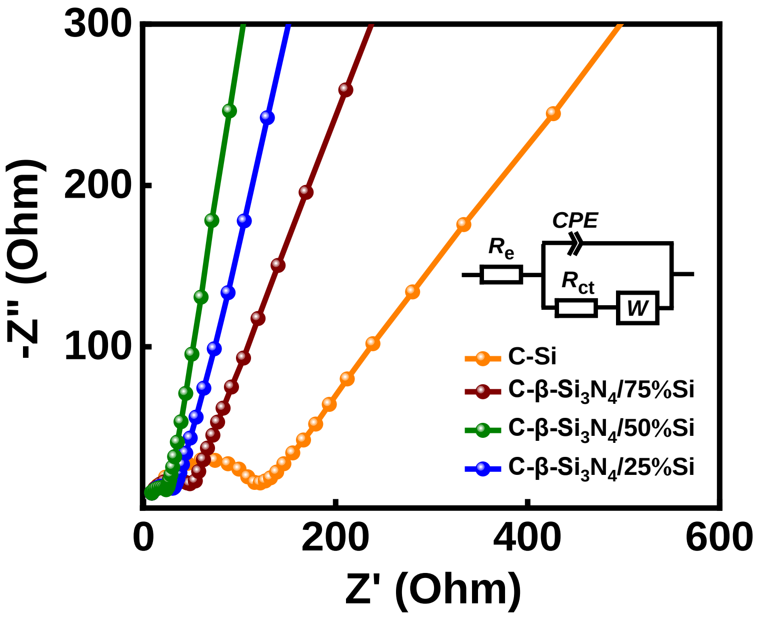

Figure S8. EIS data of various electrodes acquired after 300 charge–discharge cycles.

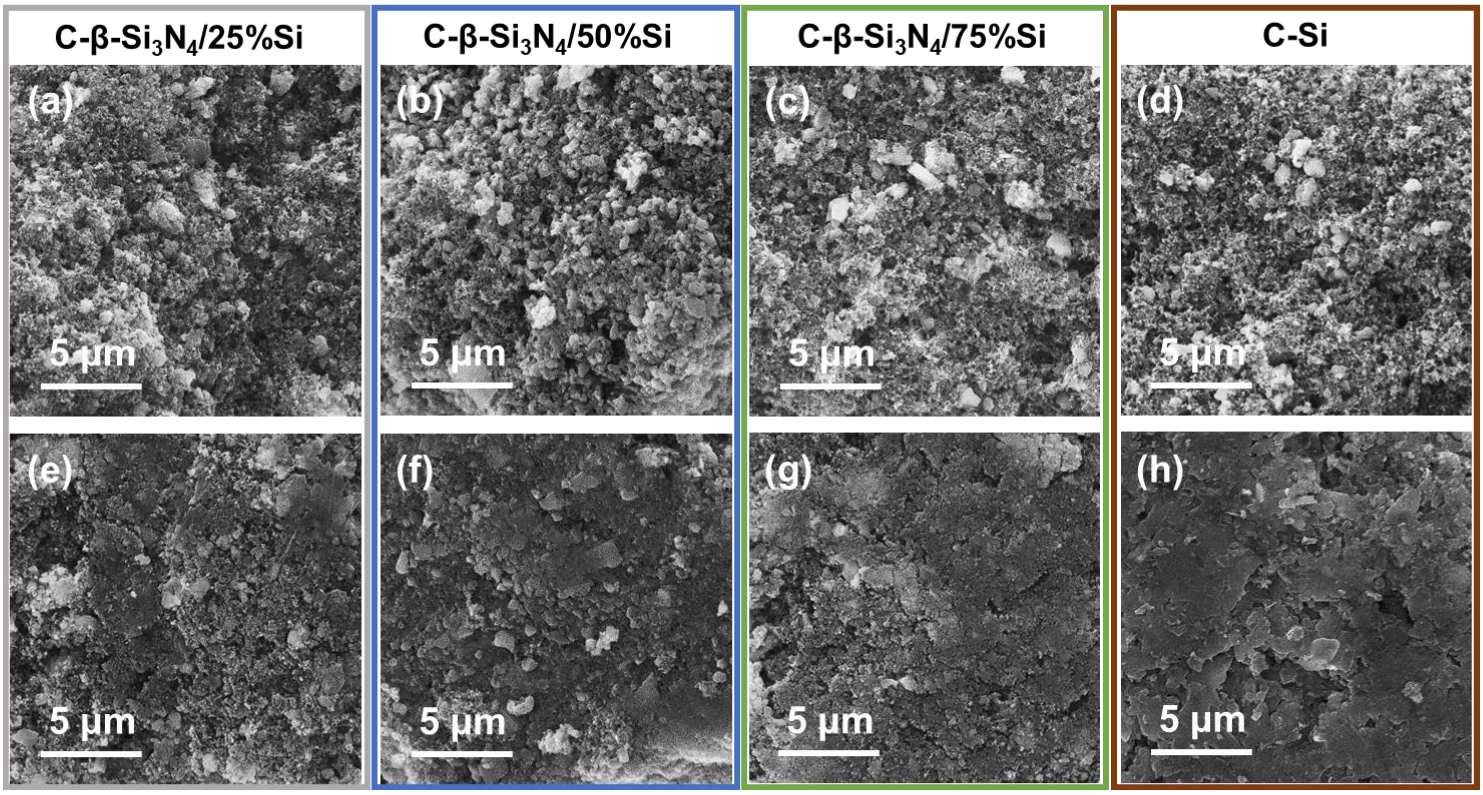

Figure S9. SEM images of C-β-Si_3N_4/25%Si, C-β-Si_3N_4/50%Si, C-β-Si_3N_4/75%Si, and C-Si electrodes (a–d) before and (e–h) after 300 charge–discharge cycles.

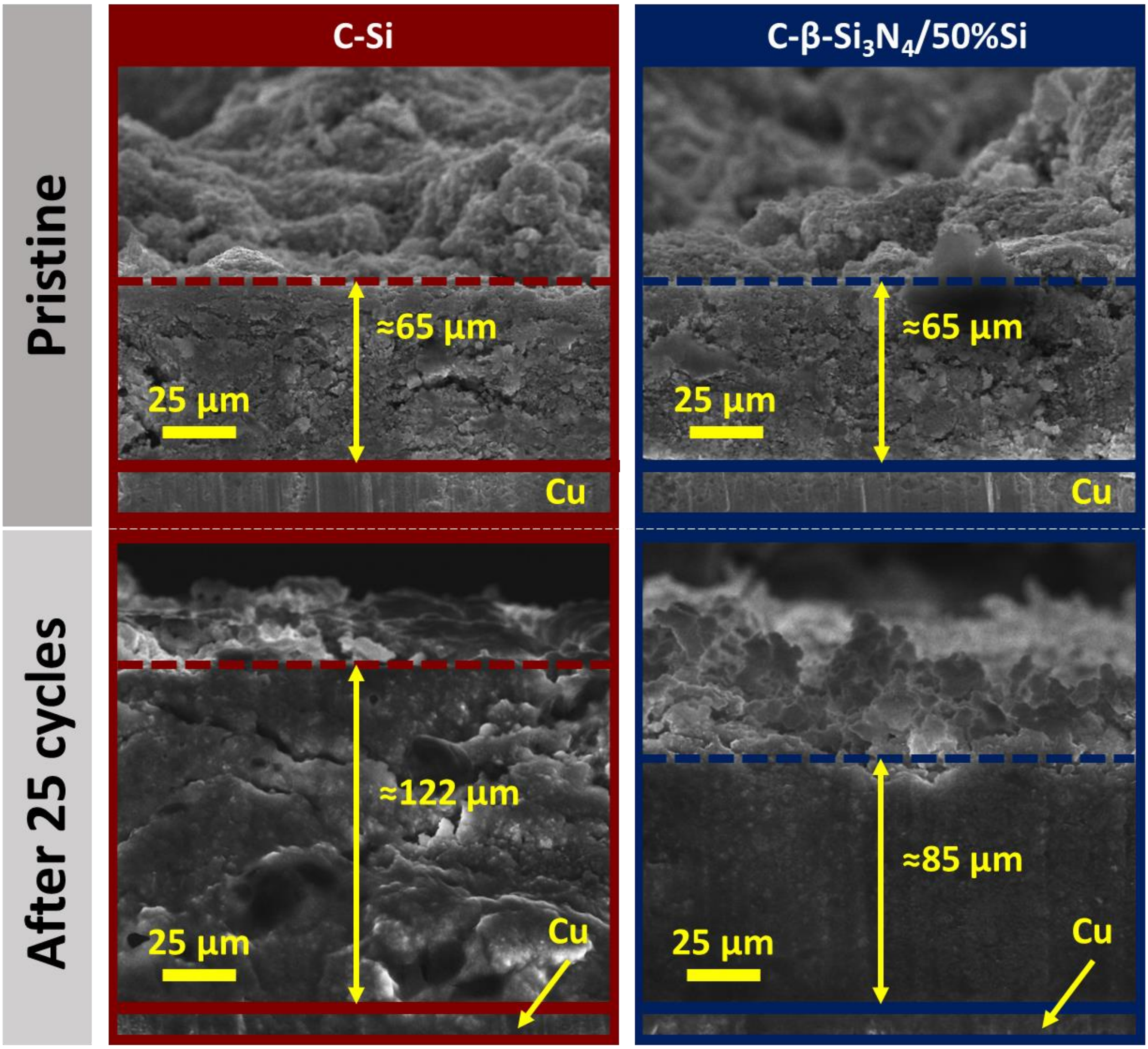

Figure S10. Cross-section SEM images of C-Si and C-β-Si_3N_4/50%Si electrodes before and after 25 charge–discharge cycles.

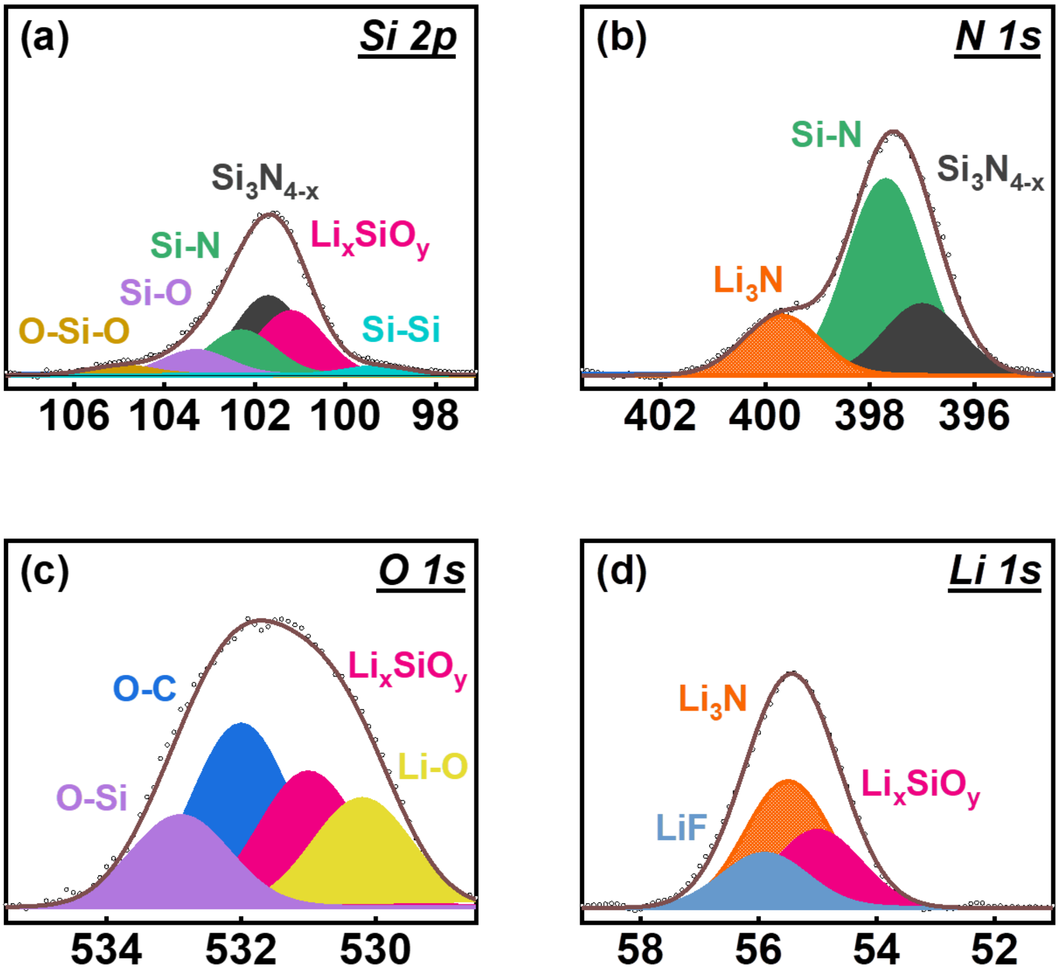

Figure S11. XPS (a) Si 2p, (b) N 1s, (c) O 1s, and (d) Li 1s spectra of C-β-Si_3N_4/50%Si electrode after 300 charge–discharge cycles.

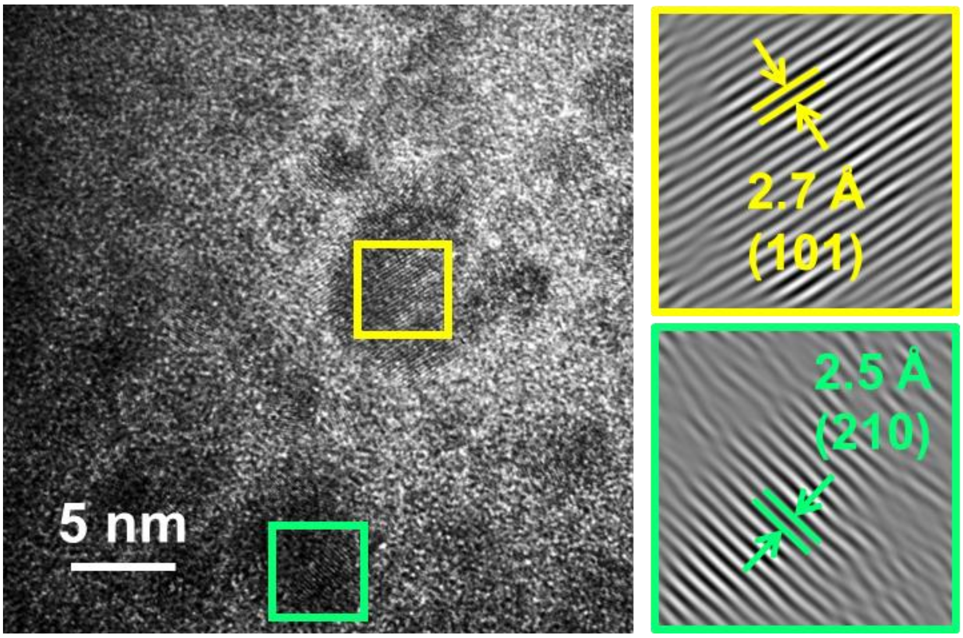

Figure S12. TEM images of C-β-Si_3N_4/50%Si sample after 300 charge–discharge cycles.

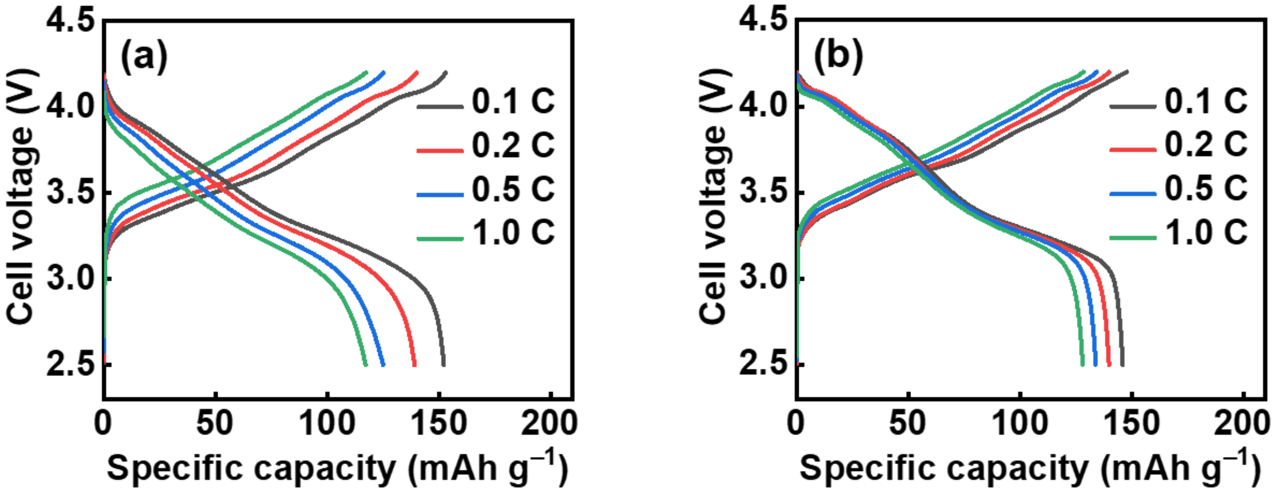

Figure S13. Charge–discharge curves of (a) C-β-Si_3N_4/50%Si||NMC-811 and (b) C-β-Si_3N_4/50%Si@AG||NMC-811 full cell measured at various rates. The specific capacities are calculated based on total mass of anode and cathode active materials.
